# Dipeptide repeat proteins inhibit homology-directed DNA double strand break repair in C9ORF72 ALS/FTD

**DOI:** 10.1186/s13024-020-00365-9

**Published:** 2020-02-24

**Authors:** Nadja S. Andrade, Melina Ramic, Rustam Esanov, Wenjun Liu, Mathew J. Rybin, Gabriel Gaidosh, Abbas Abdallah, Samuel Del’Olio, Tyler C. Huff, Nancy T. Chee, Sadhana Anatha, Tania F. Gendron, Claes Wahlestedt, Yanbin Zhang, Michael Benatar, Christian Mueller, Zane Zeier

**Affiliations:** 1 Department of Psychiatry & Behavioral Sciences, Center for Therapeutic Innovation, University of Miami Miller School of Medicine, 1501 NW 10th Ave, Biomedical Research Building Room 413, Florida, Miami 33136 USA; 2 Department of Biochemistry and Molecular Biology, University of Miami Miller School of Medicine, 1600 NW 10th Ave, Miami, FL 33136 USA; 3 John P Hussman Institute for Human Genomics, University of Miami Miller School of Medicine, 1501 NW 10th Ave, Miami, FL 33136 USA; 4 Sylvester Comprehensive Cancer Center, Biomedical Research Building, 1501 NW 10th Avenue, Miami, FL 33136 USA; 5 Department of Neurology, University of Massachusetts Medical School, Worchester, MA USA; 6 Dr. John T Macdonald Foundation Department of Human Genetics, University of Miami Miller School of Medicine, 1601 NW 12th Ave, Miami, FL. 33136 USA; 7 Department of Neuroscience, Mayo Clinic, 4500 San Pablo Rd, Jacksonville, FL 32224 USA; 8 Department of Neurology, University of Miami Miller School of Medicine, 115 NW 14th St.,, Miami, FL 33136 USA; 9 Department of Pediatrics and Horae Gene Therapy Center, University of Massachusetts Medical School, Worcester, MA USA

**Keywords:** Amyotrophic lateral sclerosis, DNA damage, DNA double strand break repair, Induced pluripotent stem cells, CRISPR, Single-strand annealing, RAD52, Homology-directed repair

## Abstract

**Background:**

The C9ORF72 hexanucleotide repeat expansion is the most common known genetic cause of amyotrophic lateral sclerosis (ALS) and frontotemporal dementia (FTD), two fatal age-related neurodegenerative diseases. The C9ORF72 expansion encodes five dipeptide repeat proteins (DPRs) that are produced through a non-canonical translation mechanism. Among the DPRs, proline-arginine (PR), glycine-arginine (GR), and glycine-alanine (GA) are the most neurotoxic and increase the frequency of DNA double strand breaks (DSBs). While the accumulation of these genotoxic lesions is increasingly recognized as a feature of disease, the mechanism(s) of DPR-mediated DNA damage are ill-defined and the effect of DPRs on the efficiency of each DNA DSB repair pathways has not been previously evaluated.

**Methods and results:**

Using DNA DSB repair assays, we evaluated the efficiency of specific repair pathways, and found that PR, GR and GA decrease the efficiency of non-homologous end joining (NHEJ), single strand annealing (SSA), and microhomology-mediated end joining (MMEJ), but not homologous recombination (HR). We found that PR inhibits DNA DSB repair, in part, by binding to the nucleolar protein nucleophosmin (NPM1). Depletion of NPM1 inhibited NHEJ and SSA, suggesting that NPM1 loss-of-function in PR expressing cells leads to impediments of both non-homologous and homology-directed DNA DSB repair pathways. By deleting NPM1 sub-cellular localization signals, we found that PR binds NPM1 regardless of the cellular compartment to which NPM1 was directed. Deletion of the NPM1 acidic loop motif, known to engage other arginine-rich proteins, abrogated PR and NPM1 binding. Using confocal and super-resolution immunofluorescence microscopy, we found that levels of RAD52, a component of the SSA repair machinery, were significantly increased iPSC neurons relative to isogenic controls in which the C9ORF72 expansion had been deleted using CRISPR/Cas9 genome editing. Western analysis of post-mortem brain tissues confirmed that RAD52 immunoreactivity is significantly increased in C9ALS/FTD samples as compared to controls.

**Conclusions:**

Collectively, we characterized the inhibitory effects of DPRs on key DNA DSB repair pathways, identified NPM1 as a facilitator of DNA repair that is inhibited by PR, and revealed deficits in homology-directed DNA DSB repair pathways as a novel feature of C9ORF72-related disease.

## Background

Despite decades of research and dozens of clinical trials, amyotrophic lateral sclerosis (ALS) remains a largely untreatable disease. There is, therefore, an urgent and unmet need for more effective therapies. The hexanucleotide repeat expansion (HRE) mutation in the chromosome 9 open reading frame 72 (C9ORF72) gene is the most common known cause of ALS and also frontotemporal dementia (FTD) [1–3]. Genetic discoveries have revealed a close relationship between ALS and FTD indicating that the development of an effective therapy for patients with C9ORF72 ALS is highly likely to benefit patients with FTD, the second leading cause of dementia. Since the initial discovery of the HRE, tremendous progress has been made in unraveling the mechanisms whereby this mutation leads to disease. While C9ORF72 haploinsufficiency may have a contributory role [4, 5], the weight of available evidence points towards toxic-gain-of-function exerted by C9ORF72 RNAs containing the expansion sequence and dipeptide repeat proteins (DPRs) that derive from non-canonical translation of the mutant transcripts [6–8].

Of the five DPRs encoded by sense and antisense C9ORF72 RNAs, proline-arginine (PR) and glycine-arginine (GR) are particularly neurotoxic as assessed in multiple model systems including induced pluripotent stem cell (iPSC) neurons, flies and rodents [6, 9–11]. Analysis of affected brain tissues also supports a role for PR and GR [12], yet their precise role in pathogenesis remains controversial [5, 13–16]. The toxicity of arginine-containing DPRs derives, in part, from their propensity to bind proteins with low complexity sequence domains that are critical for the assembly and function of the nucleolus and nuclear pore complex [16–18]. In vitro, GR and PR localize to the nucleus and disrupt pre-mRNA splicing, ribosomal RNA (rRNA) biogenesis and alter the global transcriptional program, leading to cell death [7, 9, 10]. In addition to these DPR-mediated anomalies, mutant C9ORF72 RNAs form G-quadruplex structures that engage RNA binding proteins including the abundant nucleolar proteins nucleolin (NCL) and nucleophosmin (NPM1) [19, 20]. These observations identify nuclear stress as a prominent and persistent cellular phenotype of C9ALS/FTD that has been observed in patient tissues [7, 21] and across several model systems [9, 22] including reprogrammed motor neurons [7] and a mouse model [13, 23]. While it is best-known as the site of rRNAs synthesis, the nucleolus also serves as a repository of stress response effector proteins that can be rapidly mobilized during cellular perturbations [24–27].

Nucleophosmin (also known as B23) is a multi-functional nucleolar protein [20, 21, 25, 28] that regulates nucleolar assembly and function and has been implicated in C9ALS/FTD [29–33]. The RNA and DNA binding domains of NPM1, together with nuclear localization and export signals, facilitate rRNA synthesis, processing and transport [27]. The amino-terminal portion of NPM1 allows for self-oligomerization, a conformational change that is facilitated by arginine-rich proteins such as the tumor suppressor protein p14ARF [34]. Emerging evidence suggests NPM1 participates directly in DNA damage repair in the nucleoplasm [35]. Therefore, arginine-rich DPRs that bind to NPM1 may confer toxicity in a multi-modal fashion affecting cellular processes mediated by NPM1 including rRNA biogenesis, nucleocytoplasmic transport, nucleolar function, apoptotic signaling, and DNA damage repair. Notably, NPM1 has a prominent and established role in all of these processes with the exception of DNA damage repair, for which its role remains ill-defined.

The accumulation of DNA DSBs is increasingly recognized as an emerging feature of C9ALS/FTD and also other neurodegenerative diseases [36, 37], potentially due to the unique pressure that neurons are under to maintain genomic stability [38, 39]. As a consequence of their inability to utilize homologous recombination (the preferred DNA DSB repair pathway utilized by most replicating cells), high oxygen consumption, high transcription rates and longevity, neurons must utilize elaborate DNA damage response and repair cascades to maintain genomic integrity [36]. Pathways utilized by neurons to repair DNA DSBs include non-homologous end joining (NHEJ) and homology-directed repair pathways; the latter being particularly relevant for the repair of actively transcribed DNA [40–43].. Notwithstanding this, the elucidation of the specific DNA repair pathways that are disrupted in C9ALS/FTD and the HRE product(s) that are principally involved remains incomplete. Moreover, it has not previously been established whether nucleolar dysregulation and nucleolar proteins are linked to genome instability in C9ALS/FTD. To address critical gaps in the understanding of C9ORF72-related genomic instability, we sought to determine whether neurotoxic DPRs diminish DNA DSB repair efficiency and whether NPM1 dysregulation is involved.

## Methods

### Assessment of DNA repair efficiency

The I-SceI expression plasmid and four U-2 OS cell lines carrying MMEJ, NHEJ, HR, and SSA reporter cassettes were generous gifts from Dr. Jeremy Stark at the City of Hope Medical Center. I-SceI based fluorescent reporter assays were carried out as previously described with some modifications [44]. Briefly, 200,000 cells per well were seeded in a 6-well plate. For DPR overexpression, the following day cells were co-transfected with 1 μg of I-SceI plasmid and 1 μg of PR, GR, GA or pcDNA3.1+ empty vector using Lipofectamine 2000 CD (Invitrogen, 12,566,014). For small inhibitory RNA (siRNA) knock-down experiments, cells were co-transfected with 1 μg of I-SceI and 20 μM NPM1 or control siRNAs and 1 μg of PR, GR and GA or pcDNA3.1+ empty vector using Lipofectamine 2000 CD. At 48 h after transfection, cells were washed twice with PBS, detached with 300 μL trypsin, neutralized in medium and immediately subjected to fluorescence activated cell sorting (FACS) analysis. In parallel, co-transfection experiments were carried out to identity and control for potential changes in transfection efficiency due to DPR overexpression. FACS was performed in a BD LSR-II flow cytometer, and 100,000 cells were analyzed per sample. Side and forward scatter were used to eliminate duplets and dead cells. The FCS Express 6 software suite was used for data analysis and plotting. Statistical differences between experimental groups determined by one-way ANOVA followed by post-hoc test; 6 biological replicates were used for each experimental group.

### Expression of nucleophosmin-GFP fusion proteins

Three cell lines stably expressing different GFP-NPM1 constructs were generated using plasmids obtained from Addgene (GFP-NPM1-wt (#17578), GFP-NPM1-NESΔ (#13283), GFP-NPM1-NLSΔ (#13287)). In each case, 200,000 cells were seeded into 6-well plates and transfected with 3 μg of plasmid using Lipofectamine. After 48 h, transfected cells were selected using medium containing 500 μg/mL Geneticin (ThermoFisher, 10,131,035) and propagated for 3 weeks in vitro. Cells stably expressing NPM1-GFP fusion proteins were isolated by FACS using a BD FACS SORP Aria-II.

### Quantification of nucleophosmin and PR sub-cellular localization

Each U2-OS cell line (GFP-NPM1-wt, GFP-NPM1-NESΔ, GFP-NPM1-NLSΔ) was seeded into 2 wells of a 12-well plate (80,000 cells per well) and transfected with 1 μg of PR expression plasmid using Lipofectamine. For siRNA knock-down experiments, cells were transfected with siRNAs (10 nM) targeting NPM1 or a control siRNAs with no known target using Lipofectamine 2000 CD. After 24 h, cells were fixed with 4% paraformaldehyde for 10 min, permeabilized and incubated in blocking buffer (0.2% Triton-X and 20% goat serum diluted in antibody buffer) for 40 min. Cells were washed with PBS and probed overnight with primary antibody (Anti-PR repeat, ProteinTech, 23,979–1-AP) diluted 1:500 in antibody dilution buffer. The next day, cells were washed with PBS and probed with secondary antibody at a 1:500 dilution (Alexa Fluor-594, ThermoFisher, R37117). After 2 h, cells were washed with PBS and stained with DAPI (ThermoFisher, R37605) for 5 min. Cells were imaged using the Cellomics ArrayScan high content imaging platform. Viable cells were identified using DAPI emission and threshold settings optimized for nuclear size, brightness and circularity. Automated image analysis designated sub-cellular compartments based on high DAPI emission intensity in the nucleus (circle) and low intensity in the surrounding cytoplasmic area (ring). Quantification of GFP and PR emission intensities was then computed using the circle and ring masks. The average signal intensity (distribution) throughout the nucleus and cytoplasm was ascertained by normalizing total signal intensity for each compartment to its area. To eliminate the confounding effects of high-intensity image artifacts, values exceeding 3 standard deviations above the average of all data points were removed from downstream analysis.

### Stochastic optical reconstruction microscopy (STORM) imaging

Super resolution imaging experiments were carried out with a Nikon eclipse Ti2 microscope equipped with Nikon Instruments (NSTORM). For two color dSTORM imaging, Janelia 646 and Alexa 568 secondary antibodies were used with MEA STORM imaging buffer and were imaged continuously with 10,000 frames collected per filter range at a frequency of 30 ms. The images were acquired using a 100x, 1.49 NA objective, and imaged onto a Hamamatsu C11440 ORCA-flash 4.0 camera. Storm analysis was carried out with Nikon Elements Analysis 5.02.01 for identification of molecules. Molecule list files were then exported from Nikon elements to be further analyzed using Clus-Doc software in Matlab R2018b. Cluster analysis, specifically DBSCAN function, was carried out after manually selecting regions of interest.

### iPSC culturing and neuronal differentiation

Induced pluripotent stem cells (iPSCs) were propagated and used to generate neuronal cultures as previously described [45]. Briefly, iPSCs were seeded into Matrigel coated flasks (Corning, 354,277) and supplemented with mTeSR1 medium (StemCell Technologies, 85,850). Once cultures reached ~ 80% confluency, cells were lifted using Gentle Cell Dissociation Reagent (StemCell Technologies, 07174) and transferred to low attachment (Sigma, poly-2-hydroxyethyl methacrylate, P3932) flasks containing neuronal induction media consisting of neurobasal medium (Life technologies, cat. 21,103–049) supplemented with 2% NeuroCult SM1 (StemCell Technologies, 05711), 100 ng/μl human fibroblast growth factor (bFGF; PeproTech, 100-18B), 100 ng/μl epidermal growth factor (EGF; PeproTech, AF-100-15) and 5 μg/μl heparin (Sigma-Aldrich, H3149-25KU). After two weeks, neuronal precursor cells were transferred to poly-ornithine/laminin coated flasks and cultured in neuronal maturation media consisting of DMEM/F12 (ThermoFisher, 11,320,033) supplemented with 1% N2 (Gibco, A13707–01), 2% NeuroCult SM1 (StemCell Technologies, 05711), 1% Non-essential amino acids (Gibco, 11,140–050), 2 μg/ml heparin (Sigma-Aldrich, H3149-25KU), 1% antibiotic/antimycotic, 0.1 μM Retinoic acid (Sigma-Aldrich, 302–79-4), 1 μM purmorphamine (Sigma-Aldrich, 483,367–10-8), 1 μM cAMP (Sigma-Aldrich, 60–92–4), 200 ng/ml ascorbic acid (Sigma-Aldrich, 50–81-7), 10 ng/ml glia-derived neurotrophic factor (GDNF; PeproTech, 450–10) and 10 ng/ml brain-derived neurotrophic factor (BDNF; PeproTech, 450–02). Neurons were cultured for at least 4 weeks in maturation medium prior to staining and analysis.

### Immunocytochemistry

For immunofluorescent visualization of neuronal cultures, mature neurons were gently lifted and transferred to chamber slides (MatTek, CCS-8). Following a recovery period of 3 days, neurons were fixed by exposure to 4% paraformaldehyde for 10 min, permeabilized for 15 min in 0.2% TritonX in PBS, and blocked for 40 min in 0.2% TritonX and 20% goat serum in antibody buffer. Cells were gently washed and treated with various primary antibodies overnight at 4 °C (see Additional file 1 for a full list of antibodies and dilutions used). The next day, cells were washed with PBS, probed with secondary antibodies for 2 h, washed in PBS, stained with DAPI (ThermoFisher, R37605) and mounted.

### Confocal image acquisition and analysis

Immunolabeled cells were visualized using a Zeiss LSM 710 confocal microscope with 8-bit depth, 1024 × 1024 frame size; pixel intensity values were averaged 8 times for 3 Z-stacks at 20x magnification. To quantify γH2AX and RAD52 signal intensity, values were measured for every cell within the image field with a minimum of 5 fields per biological replicate. Emission intensity values were quantified using the open source Fiji software [46]. Briefly, DAPI was used to visualize nuclei and to create an image mask in Fiji by selecting: process>binary>make binary; process>binary>watershed. The image mask was applied to the corresponding γH2AX or RAD52 images and quantified by selecting analyze>particles>analyze particles with the following threshold settings: size 10–150; circularity 0.3–1. Resulting data were transferred to MatLab to perform normalization and outlier exclusion (3 standard deviation above mean intensity). Normalized data were plotted and analyzed by one-way ANOVA or unpaired Student’s t-test using GraphPad Prism Seven.

### Protein isolation from post-mortem tissues and immunoblotting

Frozen brain tissues were homogenized in M-PER (ThermoFisher, 78,501) supplemented with phosphatase and protease inhibitors (ThermoFisher, 1,862,495 and 78,438). Quantification of proteins was performed using a BCA kit (Pierce, 23,227). For immunoblotting, 30 μg of total protein was loaded into 10% Criterion SDS gels (Bio-Rad, 5,671,034), separated by electrophoresis and transferred to nitrocellulose (Bio-Rad, 162–0232) membranes in a 20% methanol solution for 30 min at 100 V. Membranes were blocked in 5% bovine serum albumin for 1 h, and probed overnight with primary antibodies at 4 °C. After washing, membranes were probed with horse radish peroxidase conjugated secondary antibodies for 1 h. To visualize proteins of interest, substrates for enhanced chemiluminescent (ThermoFisher, 32,209) were applied to membranes for 5 min prior to X-ray film exposure and development.

### Detection of the C9ORF72 hexanucleotide repeat expansion

The presence of HREs was assessed using repeat primed PCR and capillary electrophoresis (CE) as reported previously [47]. Briefly, 30 ng of genomic DNA was amplified using FastStart PCR master mix (Sigma-Aldrich, 4,710,436,001), with 7-deaza-dGTP (New England Biolabs), Q-solution (Qiagen), DMSO, and magnesium chloride. Three primers were used: two reverse anchor primers (MRX-M13R and MRX-R1) and a forward 6FAM-fluorescent (MRX-F) labeled primer. Touchdown PCR was performed by gradually decreasing the temperature from 70 °C to 56 °C in 2 °C increments and 3-min extensions. The amplified product was denatured in Hi-Di formamide and Liz-6000 size ladder then resolved using an ABI3730 DNA Analyzer and the data processed using GeneMapper software.

Primers:

MRX-F FAM- 5′-TGTAAAACGACGGCCAGTCAAGGAGGGAAACAACCGCAGCC-3′,

MRX-13R 5′-CAGGAAACAGCTATGACC-3′,

MRX-R1, 5′-CAGGAACAGCTATGACCGGGCCCGCCCCGACCACGCCCCGGCCCCGGCCCCGG-3′.

### CRISPR/Cas9 Adeno-associated viral vectors

Four 21-nucleotide gRNAs were designed to excise the HRE where C9gR-1 and C9gR-2 target the 5′ end of the repeat and C9gR-3 and C9gR-4 flank the 3′ end of Exon 2. These guides were cloned in pairs into the 3′ end of a CB-GFP encoding AAV proviral plasmid, each under the control of a U6 promoter. Two plasmids pAAV-CB-GFP-C9gR-2-3 and pAAV-CB-GFP-C9gR-2-4 (most efficient at editing HEK293 cells) were used to package pseudotyped rAAV9 vectors. For the AAV9-pU1a-SpCas9-RBGpA vector, SpCas9 is under the control of a U1a promoter with a HA tag and a nuclear localization signal at its 5′ end while a 3′ rabbit beta-globin polyadenylation signal serves as a termination signal.

### Detection of genome editing

To detect CRISPR/Cas9 mediated editing of the C9ORF72 HRE, 25 ng of genomic DNA was amplified using forward C9var1 (AAAGAGAGGTGCGTCAAAC) and reverse C9ln1 (GGGAAAGTAAAAATGCGTCG) primers. The reaction was prepared in a 25 μL volume, consisting of 5 μL 5X Q5 buffer (New England BioLabs Inc.), 0.5 μL 10 mM dNTPs, 0.25 μL Q5 polymerase, and 5 μL 5 M Betaine. Amplification was performed using the thermocycling parameters: 1 cycle: 3 min, 98 °C; 35 cycles: 30 s, 98 °C, 20 s, 63 °C, 45 s, 72 °C; 1 cycle: 7 min, 72 °C. Resulting amplicons were resolved by electrophoreses and visualized with ethidium bromide and ultraviolet light.

### Dot blot and immunolabeling of proline-arginine

Nuclear and cytoplasmic protein fractions were isolated using the NE-PER kit (ThermoFisher, 78,833) and applied to nitrocellulose membranes using a vacuum manifold then blocked with 5% dry milk dissolved in TBS-T for 1 h. Membranes were then probed with primary antibodies (See Additional file 1 for a list of antibodies and dilutions) overnight at room temperature. The next day, membranes were washed with TBS-T, probed with secondary antibodies for 1 h at room temperature, washed for 15 min, then visualized using chemiluminescence and X-ray film as described above.

### Bisulfite pyrosequencing

Genomic DNA (gDNA) was extracted from iPSC neurons using DNeasy Blood & Tissue kit (Qiagen, 69,506); 2 μg of gDNA was treated with bisulfite and analyzed by pyrosequencing (EpigenDx Inc.) as previously reported [48]. Levels of cytosine methylation were assessed using custom assays developed by EpigenDx (ADS3232-FS1 and ADS3232-FS2) on the PSQTM96HS system. In total, methylation levels at 16 cytosine residues within the C9ORF72 promoter from − 55 to + 125 base pairs relative to the C9ORF72 transcriptional start site were quantified.

### Immunoassay analysis of poly(GP)

Levels of poly(GP) in human brain tissue lysates were measured using a Meso Scale Discovery-based immunoassay and a MSD QUICKPLEX SQ120 instrument. A purified mouse monoclonal poly(GP) antibody was used as both the capture and detection antibody (TALS 828.179, Target ALS Foundation); the capture antibody was biotinylated and used to coat a 96-well MSD Small Spot Streptavidin plate, whereas the detection antibody was tagged with an electrochemiluminescent label (MSD GOLD SULFO-TAG). Frozen brain tissues were homogenized in M-PER (ThermoFisher, 78,501) supplemented with phosphatase and protease inhibitors (ThermoFisher, 1,862,495 and 78,438). Quantification of protein concentrations in each sample was performed using a BCA kit (Pierce, 23,227). Lysates were diluted to the same protein concentration, and each sample was tested in duplicate. For each well, the intensity of emitted light, which is reflective of poly(GP) levels and presented as arbitrary units, was acquired upon electrochemical stimulation of the plate. The experimenter was blinded to the disease status of all samples.

## Results

### Nucleophosmin associates with the proline-arginine dipeptide repeat protein in multiple cellular compartments

**Fig. 1:**
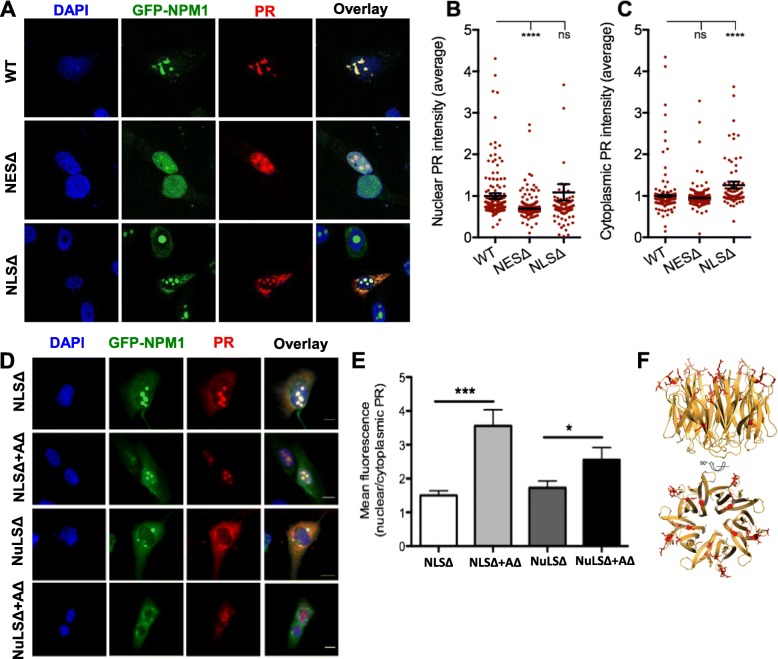
Subcellular localization of PR. **a**) Representative confocal images of U-2 OS cells stably expressing wild-type NPM1 (WT), NPM1 with deletions of the nuclear export (NESΔ) or nuclear localization (NLSΔ); NPM1 proteins were fused to GFP (green). Each NPM1 construct was co-transfected with PR (red) and nuclei stained with DAPI (blue). Automated microscopy and image analysis was used to quantify levels of PR in the nucleus (**b**) or cytoplasm (**c**) as a function of area (average intensity). Statistical significance was assessed by one-way ANOVA and post-hoc test between each experimental group; *n* = 2 biological groups, 9 fields per group, ****P < 0.0001; error bars are SEM, dots represent single cells. **d**) Representative images of U-2 OS cells stained with DAPI (blue) expressing GFP-NPM1 fusion proteins (green) with deletions of the nuclear localization (NLSΔ) or nucleolar (NuLSΔ) signals to confer cytoplasmic localization. Cells were immunolabeled with an antibody against PR (red). **e**) Nuclear/Cytoplasmic mean fluorescence (Y-axis) for *n* > 30 cells per mutant cell line (X-axis), *n* = 3 biological replicates. *P < 0.05; ***P < 0.0005 student’s t-test; error bars are SEM. **f**) Cartoon (created in PyMOL) with top and side perspectives of the NPM1 (PDB 4N8M) pentamer

When expressed in vitro, PR localizes within nucleoli [9, 10, 22, 49], presumably due to contacts between the basic arginine residues of PR and acidic residues of NPM1 [50, 51]. Here, we sought to determine whether binding between PR and NPM1 is dependent upon the nucleolar micro-environment or if their interaction is independent of sub-cellular localization. To test this, we used expression plasmids to generate cell lines that stably express three different GFP-NPM1 fusion proteins: one with an unmodified NPM1 sequence (Addgene 17,578, wild-type (WT)), a nuclear localization signal deletion mutant (Addgene 13,787, NLSΔ) or a nuclear export signal deletion mutant (Addgene 13,283, NESΔ). As expected, the NPM1 NESΔ mutant localized within nucleoli and the nucleoplasm, but not the cytoplasm. The NPM1 NLSΔ mutant was detectable primarily in nucleoli and the cytoplasm while unmodified (WT) NPM1 localized almost exclusively within nucleoli (Fig. 1a). When co-expressed with PR, we found that the sub-cellular localization of PR mirrored that of each NPM1 construct. Automated microscopy and image analysis revealed that the nuclear PR intensity, relative to nuclear area (average intensity), was significantly decreased (one-way ANOVA: F_(2,957)_ = 15.19, *P* < 0.0001) in NESΔ NPM1 expressing cells (Fig. 1b). This unbiased analysis confirms that PR is more evenly distributed between the nucleolus and nucleoplasm when co-expressed with NESΔ NPM1. When co-expressed with NLSΔ NPM1, cytoplasmic levels of PR were significantly increased (one-way ANOVA: F_(2,956)_ = 29.32, *P* < 0.0001), suggesting a direct interaction between NLSΔ and PR in the cytoplasm (Fig. 1c). To confirm this, we isolated nuclear and cytoplasmic proteins from cells expressing PR and either WT NPM1 or NLSΔ NPM1. A dot-blot analysis revealed a significant increase in cytoplasmic PR levels when co-expressed with NLSΔ NPM1, as compared to co-expression with WT NPM1 (Additional file 2D). These results strongly suggest that a direct interaction occurs between PR and NPM1 across cellular compartments and that inhibition of NPM1 by PR occurs globally (Fig. 1a).

Previously, it was found that substituting specific acidic residues of NPM1 prevents binding between NPM1 and basic arginine residues of the tumor suppressor protein p14-ARF [50, 51]. Assuming these acidic residues are also necessary for the interaction between NPM1 and the arginine residues of PR, we substituted six acidic residues of NPM1 (D34G, D36G, E37G, E39G, E61G, E93G), hereafter referred to as the acidic loop deletion (AΔ). Next, we generated three additional GFP-NPM1 constructs: a nucleolar localization mutant (GFP-NPM1-NuLSΔ), a NuLSΔ and AΔ double mutant (GFP-NPM1-NuLSΔ+AΔ) and an NLSΔ and AΔ double mutant (GFP-NPM1-NLSΔ+AΔ). The cytoplasmic co-localization of PR and NPM1 was significantly reduced by the deletion of the NPM1 acidic loop (unpaired student’s t-test: GFP-NPM1-NLSΔ+AΔ, *P* < 0.005; GFP-NPM1-NuLSΔ+AΔ, *P* < 0.05) (Fig. 1d,e). Furthermore, unbiased high-content image analysis revealed a modest but significant decrease in the levels of PR within nucleoli when NPM1 levels were depleted using a small inhibitory RNA (siRNA) (unpaired student’s t-test: *P* < 0.05) (Additional file 2B). The results of these experiments provide additional evidence that PR binds directly to NPM1 and show, for the first time, that this interaction is independent of complex liquid-liquid phase dynamics that govern nucleolar assembly and organization [16]. Therefore, the multiple functions of NPM1 occurring both within nucleoli and other cellular compartments are likely to be impaired by PR.

### Dipeptide repeat proteins reduce the efficiency of multiple double strand DNA break repair pathways

**Fig. 2:**
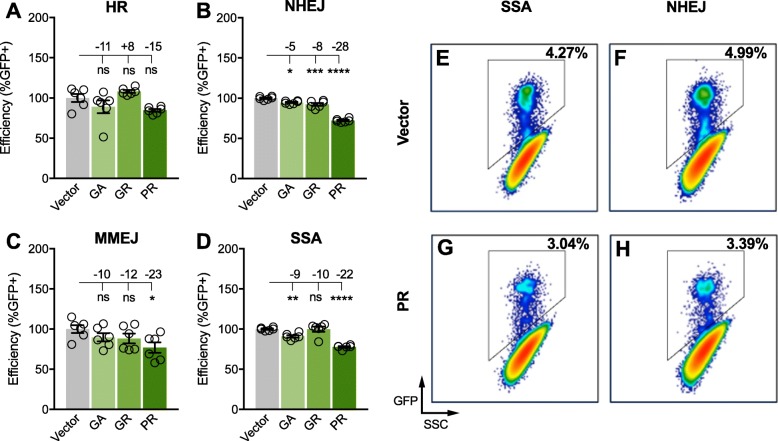
Efficiency of double strand DNA break repair pathways in response to dipeptide repeat proteins. **a**-**d**) Relative repair efficiency (Y-axis) as determined by the percentage of GFP positive cells in cultures transfected with DPR expression plasmids or an empty vector (set to 100% efficiency). Four reporter cell lines were used to assess the efficiency of (**a**) homologous recombination (HR), (**b**) non-homologous end joining (NHEJ), (**c**) microhomology mediated end joining (MMEJ), and (**d**) single strand annealing (SSA). Statistical significance was assessed by one-way ANOVA and post-hoc test between each experimental group and the control group (vector); *n* = 6 biological replicates, 100,000 cells/replicate were evaluated; error bars are SEM, *p < 0.05, ***p < 0.0005, ****p < 0.0001. Inset numbers are the mean difference between groups. E-H) Representative fluorescence activated cell sorting plots of transfected NHEJ and SSA reporter cells using GFP fluorescence (Y-axis) and side scatter (X-axis); the number of GFP positive cells is represented as a percentage of parent gating

To investigate the role of DPRs in DNA DSB repair pathways, we used a pathway-specific reporter system as previously described in detail [44]. Briefly, cell lines were engineered to have inactive GFP cassettes with recognition sequences for the rare-cutting meganuclease I-SceI. Expression of I-SceI in these cells causes DNA DSBs to occur within the GFP cassettes. Due to the design of each cassette, GFP expression will occur only if the intended repair pathway is utilized, thus repair efficiency can be represented by the proportion of GFP expressing cells that are identified by fluorescence activated cell sorting (FACS). For the detection of homology-directed DSB repair events, the removal of non-homologous insertion sequence is required for the restoration of GFP expression. For the detection of end-joining events, tandem I-SceI sites were placed within a GFP cassette, therefore, restoration of GFP does not require sequence homology. Here, four cell lines, each with a unique pathway-specific DNA DSB reporter cassette were co-transfected with the I-SceI expression plasmid (Addgene ID 44026, EJ5GFP) and one synthetic DPR plasmid expressing GA, GR, PR or an empty control vector. After 72 h, transfected cells were analyzed by FACS to quantify the number of GFP positive cells. We found that the efficiency of NHEJ (− 8%, *P* < 0.0001), but not other pathways, was reduced by GR overexpression (Fig. 2) while over-expression of PR had pronounced inhibitory effects on NHEJ efficiency (− 28%, *P* < 0.0001), micro-homology mediated end joining (MMEJ) (− 23%, *P* < 0.05), and SSA (− 22%, *P* < 0.0001) (Fig. 2). Representative FACS scatter plots show that the raw percentage of GFP positive cells after gating was reduced in PR transfected cells by 1.23% (4.27–3.04%) and 1.6% (4.99–3.39%) for SSA and NHEJ, respectively (Fig. 2e-h). The efficiency of homologous recombination (HR) was affected by the overexpression of DPRs (one-way ANOVA: F_3,20_ = 4.836, *P* = 0.0109), but a post-hoc analysis found no significant differences between experimental groups and the control group (Fig. 2a). In contrast, post-hoc analyses found the efficiency of NHEJ (− 5%, one-way ANOVA: F_3,20_ = 104.6, *P* < 0.0001) and SSA (− 9%, one-way ANOVA: F_3,20_ = 30.76, *P* < 0.0001) were significantly reduced by GA, but the magnitude of inhibition was modest (Fig. 2b,d). To ensure that expression of DPRs does not interfere with transfection of the I-SceI expression plasmid, which could confound our analysis, we performed a co-transfection control experiment whereby U2-OS cells were transfected with a GFP expression plasmid and each DPR expression plasmid. We observed no significant difference in the number of GFP expressing cells across the experimental groups, suggesting that changes in transfection efficiency cannot explain our finding that DPRs reduce the efficiency of DNA DSB repair (Additional file 3).

### Nucleophosmin facilitates single strand annealing and non-homologous end joining

**Fig. 3:**
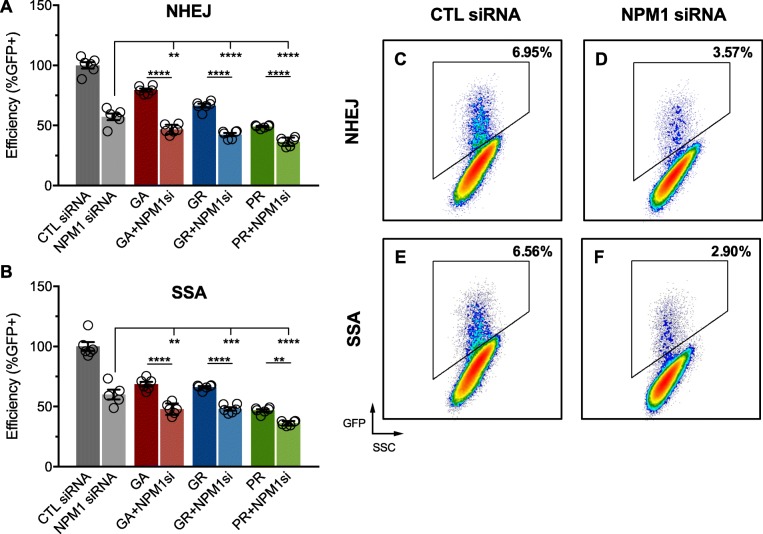
DNA repair efficiency in response to the manipulation of nucleophosmin levels. **a**-**b**) Repair efficiency (Y-axis) as determined by the percentage of GFP positive cells in cell cultures transfected with proline-arginine (PR), glycine-arginine (GR), glycine-alanine (GA) and nucleophosmin siRNA, or control siRNA. Two reporter cell lines were used to assess the efficiency of (**a**) non-homologous end joining (NHEJ) and (**b)** single strand annealing (SSA). Statistical significance was assessed by one-way ANOVA and post-hoc test between each experimental group and the control group (control siRNA); n = 6 biological replicates, 100,000 cells/replicate were evaluated; error bars are SEM, *p < 0.05, ***p < 0.0005, ****p < 0.0001. (**c**-**f**) Representative fluorescence activated cell sorting plots of transfected NHEJ and SSA reporter cells using GFP fluorescence (Y-axis) and side scatter (X-axis); the number of GFP positive cells is represented as a percentage of parent gating

Previous studies have shown that expression of PR increases the frequency of DNA DSBs in cells [52] and that NPM1 facilitates DNA repair [3, 35, 52], but it is unknown whether PR confers its genome destabilizing effects through inhibition of NPM1. Therefore, we sought to determine whether the depletion of NPM1 would increase the frequency of DNA DSBs and exacerbate the effects of PR on DNA DSB repair efficiency. While NPM1 has been generally implicated in DNA break repair [35, 53], no previous examinations of its role in specific DNA DSB repair pathways has been reported. We found that depletion of NPM1 with a siRNA significantly reduced the efficiency of NHEJ (one-way ANOVA: F _[_7_,_^40^_]_=143, *P* < 0.0001) and SSA (one-way ANOVA: F _[_7_,_^39^_]_=82.98, *P* < 0.0001) (Fig. 3). The effects of NPM1 depletion were more pronounced in DPR-expressing cells when compared to NPM1 siRNA or DPR-expression alone. Efficient NPM1 depletion and PR expression was confirmed by RT-PCR, western blot, and dot blot (Additional file 4). These results suggest a role for NPM1 in DNA DSB repair and that PR and GR may inhibit these pathways through an NPM1-dependent mechanism. In support of this notion, GR and PR are known to bind to NPM1 directly [9, 49]. However, since GA does not bind to NPM1 but had a similar additive inhibitory effect on DNA DSB repair, the observation may simply indicate that DPRs and NPM1 depletion inhibit DNA repair through independent mechanisms.

Next, we transfected U-2 OS cells with PR, an NPM1 siRNA, or a combination of both and used immunocytochemistry (ICC) to quantify levels of γH2AX, a well-established marker of DNA damage, commonly associated with DNA DSBs. Expression of PR and depletion of NPM1 significantly increased DNA DSB frequency relative to un-transfected cells, or cells transfected with an inactive siRNA (Additional file 5A). Depletion of NPM1 alone enhanced DNA double strand break frequency following exposure to etoposide, further suggesting a role for NPM1 in DNA DSB repair (Additional file 5B).

### Nucleophosmin translocates to sites of single strand annealing and interacts with the double strand DNA break repair machinery

**Fig. 4:**
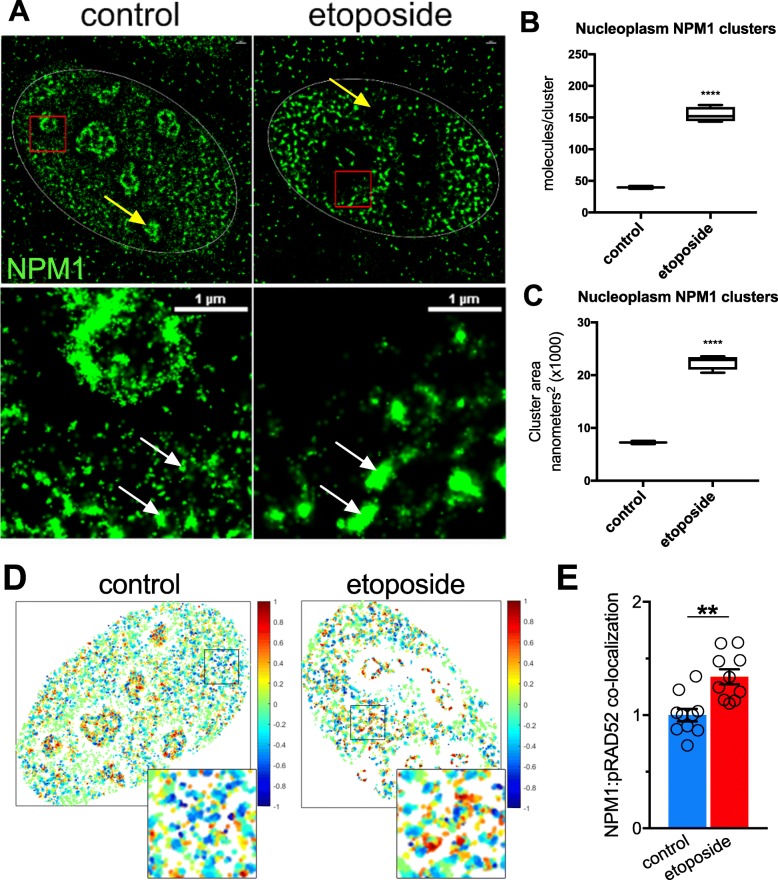
Super-resolution Stochastic Optical Reconstruction Microscopy (STORM) shows nuclear co-localization of nucleophosmin and phospho-RAD52. **a**) Representative images of NPM1 immunostaining (green) in the nuclei (white oval traces in top panels) of cells treated with etoposide or vehicle control. Red boxes indicate area of increased magnification in bottom panels; yellow arrows indicate nucleoli; white arrows indicate NPM1 clusters. **b**-**c**) Quantification of NPM1 clustering within the nucleoplasm of 3 cells with and without etoposide treatment. ****P < 0.0001, as determined by unpaired student’s t-test, error bars are SEM. **d**) Representative super-resolution analysis of U2-OS cells treated with etoposide to induce DNA DSBs or vehicle control then stained with antibodies against NPM1 and pRAD52. Colored heat-map where red indicates positive spatial overlap of NPM1 and pRAD52 (correlation coefficient *r* = 1) and blue indicates negative correlation (*r* = − 1). E) Numerical quantification of NPM1 and pRAD52 co-localization in the nucleus, *n* = 10 cells for each condition. Significance was assessed by unpaired student’s t-test (**P < 0.01); error bars are SEM

A hallmark of nuclear stress is nucleolar disassembly and dispersal of nucleolar proteins [9, 10, 54]. Previous studies suggest that cellular stress leads to the dissociation of NPM1 pentamers in nucleoli and recruitment of monomeric NPM1 to sites of DNA damage and repair [27, 35, 53]. We found that NPM1 affects SSA and NHEJ efficiency (Fig. 3) and sought to determine whether inducing DNA DSBs results in the translocation of NPM1 from the nucleolus to nucleoplasm. To this end, U-2 OS cells were treated with etoposide (200 μM) for 1 h to induce DNA DSBs. Using super-resolution Stochastic Optical Reconstruction Microscopy (STORM) [55, 56], we found that levels of γH2AX were robustly increased by exposure to etoposide, confirming efficient induction and detection of DNA damage (Additional file 6). Using an antibody against NPM1, we then assessed NPM1 sub-cellular localization with nanometer accuracy. We found that levels of NPM1 were dramatically reduced within nucleoli of cells treated with etoposide (Fig. 4a), whereas an analysis of the nucleoplasm revealed a highly significant increase in both the number of NPM1 molecules/cluster and cluster area, indicating disassembly of the nucleolus and translocation of NPM1 from the nucleolus to nucleoplasm (Fig. 4b,c). Next, we sought to determine whether NPM1 associates with phosphorylated RAD52 (pRAD52), a key mediator of SSA. In mammalian cells, phosphorylation of RAD52 at residue Y104 increases its affinity for single stranded DNA and facilitates the annealing of complementary DNA strands during SSA [57]. We found that exposure to etoposide increased co-localization of γH2AX and pRAD52, indicating efficient activation of the SSA pathway (Additional file 6B). Moreover, we found that etoposide significantly increased (student’s t-test: *P* < 0.01) the degree of NPM1 and pRAD52 co-localization in the nucleus of U-2 OS cells, further supporting a role for NPM1 in SSA (Fig. 4d,e).

### DNA DSB repair proteins are elevated in C9ALS iPSC neurons but not in those with a hypermethylated C9ORF72 promoter

Having observed that PR reduced the efficiency of NHEJ and SSA, we sought to determine whether these pathways are dysregulated in a more relevant cell type. We generated iPSC motor neurons (iMNs) using two previously-characterized C9ALS iPSC lines (C9ALS-1, C9ALS-2) from our unique patient population [48] and quantified levels of a general marker of DNA damage foci (γH2AX), a marker of NHEJ (Ku-70) and SSA (pRAD52). Quantification of γH2AX levels by western analysis revealed a significant increase after 60 days of motor neuron differentiation for both cell lines as compared to motor neurons derived from two unaffected control cell lines (Additional file 7A). There were also increased levels of Ku-70 (Additional file 7B) and a dramatic increase in the level of pRAD52 [58] for C9ALS-1 iMNs but not the C9ALS-2 iMNs (Additional file 7C). Previously, we found that levels of DNA methylation at the C9ORF72 promoter varies during cellular reprograming of C9ALS patient cell lines and in the brain of transgenic mice that harbor the human HRE expansion [48, 59]. To determine whether epigenetic repression of the C9ORF72 promoter might explain why the C9ALS-1 iMNs, had a more pronounced enrichment of DNA DSB markers as compared to C9ALS-2 iMNs, we used bisulfite pyrosequencing to quantify the level of cytosine methylation across 16 CpG dinucleotides near the C9ORF72 transcriptional start site. Mean CpG methylation levels were 17.8 and 57.4% for C9ALS-1 and C9ALS-2 iMNs, respectively (Additional file 7D). This suggests that epigenetic repression of the C9ORF72 promoter leading to reduced transcription and DPR production likely accounts for the reduced levels of DNA DSB markers in C9ALS-2 iMNs. Epigenetic repression of the C9ORF72 locus has been shown to alter C9ORF72 RNA splicing, DPR production and features of the C9ALS/FTD clinical presentation [60–62]. While based on only two cell lines, our results suggest C9ORF72 promoter hypermethylation alters DNA damage phenotypes in C9ALS iPSC neurons as well.

### Genome editing of induced pluripotent stem cells eliminates the C9ORF72 hexanucleotide expansion and DPR expression in neurons

Having observed variable DNA damage phenotypes across iPSC cell lines, we sought to excise the C9ORF72 expansion mutation and generate isogenic control cell lines. To this end, C9ALS iPSCs were transduced with recombinant adeno-associated viral (AAV) vectors that express the Cas9 endonuclease and guide RNAs (gRNAs) flanking the C9ORF72 expansion. After 2 weeks, iPSC cultures were dissociated, titrated and seeded at low density. Clonal iPSC colonies were then manually selected, expanded and screened using end-point PCR with primers spanning the expansion mutation and gRNA recognition sequence to detect editing events. With this strategy, we selected iPSC clones from one patient cell line (C9ALS-1) for which a shift from 521 to 321 base pairs was observed - indicating that genomic editing had occurred (Additional file 8A). Another technique, repeat primed PCR, was used to amplify the HRE sequence directly. This analysis confirmed that deletion of the expansion had occurred for one of the clones (C9ALS-1.11; hereafter referred to as C9ALS-1 iso) but not two other clones (C9ALS-1.4 and C9ALS-1.8) (Additional file 8A). We obtained two additional patient-derived iPSC cell lines from the Cedars-Sinai iPSC core facility (CS29iALS-C9nxx, CS52iALS-C9nxx; hereafter referred to as C9ALS-4 and C9ALS-5) and corresponding isogenic controls (CS29iALS-C9n1.ISOxx, CS52iALS-C9n6.ISOxx; hereafter referred to as C9ALS-4 iso and C9ALS-5 iso) that were generated using similar genome editing methods. We used repeat-primed PCR to confirm the presence of the HRE in these patient derived iPSC lines and lack of the mutation in isogenic controls (Additional file 8B). To confirm that genome editing eliminates DPR expression in isogenic control lines, we generated cultures of iPSC neurons. As expected, PR expression was evident in Tuj1 positive neurons harboring the expansion (HRE+), but not HRE- isogenic controls (Additional file 8C). To further confirm efficient neuronal differentiation, cultures were stained with ISL-1, a motor neuron marker and NeuN, a neuronal nuclear envelope protein (Additional file 9).

### The single strand annealing repair pathway is constitutively activated in C9ALS iPSC motor neurons and C9ALS/FTD brain tissues

**Fig. 5:**
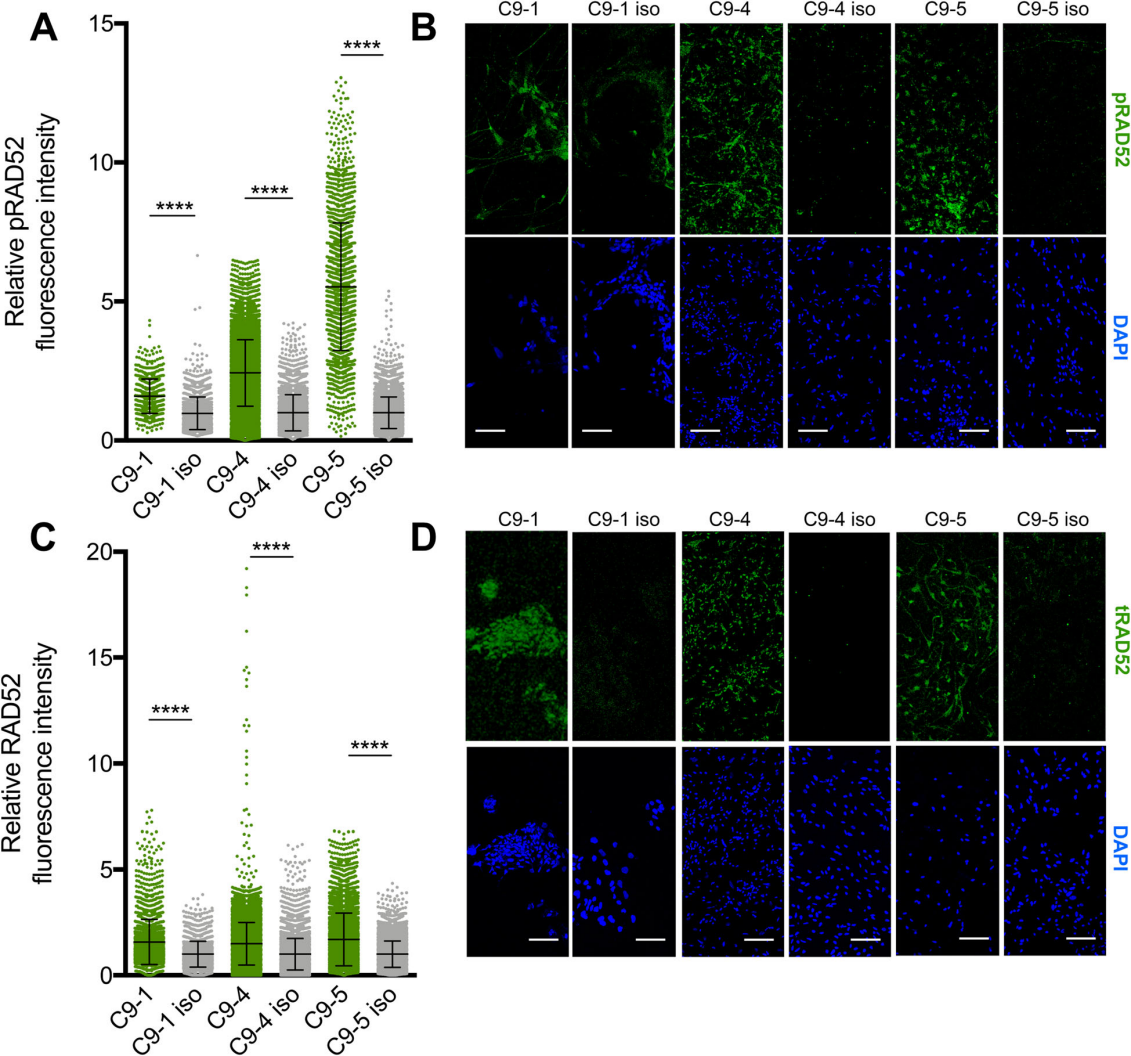
Expression of activated and total RAD52 in C9ALS/FTD neurons. **a**) Quantification of pRAD52 mean fluorescence in neurons using automated image analysis software (Fiji/Image J) for three C9ALS iPSC lines normalized to isogenic lines; each data point represents one cell. **b**) Representative confocal immunofluorescence images of iPSC motor neuron cultures stained with an antibody against phospho-RAD52 (pRAD52) (green) and counter-stained with DAPI (blue) scale bars are 100 μm. **c**) Quantification of total RAD52 (tRAD52) mean fluorescence normalized to isogenic line. **d**) Representative images of iPSC motor neuron cultures immuno-stained with an antibody against RAD52 (tRAD52) (green) and DAPI (blue). n = 3 biological replicates, 5 fields per replicate, error bars are SEM; ****P < 0.0001, as determined by unpaired student’s t test

Semi-automated image analysis of C9ALS iMNs derived from iPSC cell lines with unmethylated C9ORF72 promoters (C9ALS-1, C9ALS-4, C9ALS-5) and their isogenic controls revealed significantly higher levels of γH2AX (unpaired student’s t-test: *P* < 0.0001) relative to isogenic iMNs (Additional file 10). To investigate whether reduced SSA efficiency might contribute to this accumulation of DNA DSBs in HRE+ iPSC neurons, we quantified levels of pRAD52 and total RAD52 (tRAD52). Relative to isogenic control lines, HRE+ iPSC neurons had increased levels (unpaired student’s t-test: *P* < 0.0001) of pRAD52 (Fig. 5a,b) and also tRAD52 (Fig. 5c,d).

**Fig. 6:**
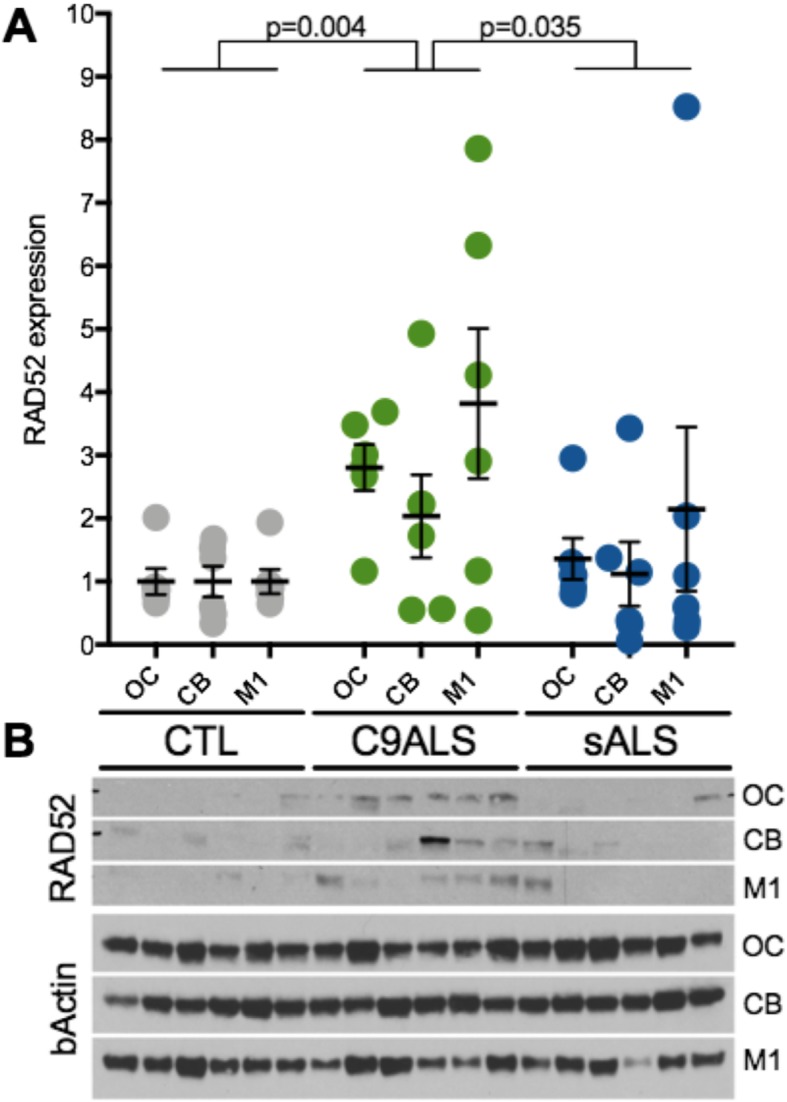
Quantification of total RAD52 in human brain samples. **a**) Western blot quantification of total RAD52 from unaffected controls (CTL), C9ORF72-related ALS (C9ALS) and sporadic ALS (sALS) in three different brain regions: Occipital cortex (OC) Cerebellum (CB) and Motor cortex (M1). Comparisons between diagnosis groups were performed by mixed effect analyses utilizing data from all three brain regions and accounting for both the between-region differences and within-person correlation; n = 6 per diagnosis group, 3 measurements per person, one from each region. **b**) Representative western for total RAD52 and beta-actin

To determine whether PR-mediated inhibition of SSA efficiency (Fig. 2) and hyperactivation of SSA in HRE+ iPSC neurons (Fig. 5) is indicative of SSA dysregulation in the brain of C9ALS/FTD patients, we isolated protein from three regions of post-mortem tissues: motor cortex, occipital cortex, or cerebellum (sample identifiers and demographic information are summarized in Additional file 11). In a comparison between diagnosis groups, we observed increased RAD52 levels in C9ALS samples for all three brain regions (Fig. 6). In a mixed effect analysis accounting for between-region and within-person correlation, a significant increase in RAD52 levels was observed for C9ALS samples as compared to unaffected controls (*p* = 0.004) and sALS (*p* = 0.035). In a brain-region-specific analysis, increased RAD52 levels in C9ALS samples reached significance for the occipital cortex compared to unaffected controls (one-way ANOVA, *p* = 0.0023) and sALS (one-way ANOVA, *p* = 0.0119), but not other regions due to a high degree of variability among samples (Additional file 12). Levels of activated pRAD52 were also highly variable across the tissue cohort (Additional file 12), likely due to aberrant phosphatase activity that is known to occur in post-mortem tissues [63, 64]. Similarly, levels of 53BP1 were highly variable and did not statistically differ between diagnosis groups (Additional file 12). To confirm expression of DPRs in brain tissue lysates from individuals in the clinical cohort, a Meso Scale Discovery-based immunoassay was used to measure glycine-proline DPRs [poly(GP)] in each brain region and diagnosis group. Poly(GP), one of the more abundant DPRs in C9ALS brain tissue [15, 65–68], is produced by repeat associated non-ATG translation of both the sense and anti-sense HRE transcripts. Consistent with prior findings, poly(GP) levels were significantly higher in C9ALS patient tissues compared to CTL and sALS samples, with levels being the most pronounced in the cerebellum (Additional file 13) [65, 69].

## Discussion

Nucleolar dysfunction is recognized as a key feature of C9ORF72-related neurodegeneration and, as a consequence of this dysfunction, various inter-related cellular processes are affected [13]. While there is substantial evidence supporting a role for dysregulated RNA metabolism and nucleocytoplasmic transport defects in C9ALS/FTD [10, 16], accumulating evidence supports a role for aberrant DNA DSB repair as well [52, 70–73]. Since the nucleolus is a repository of stress response proteins, and nucleolar proteins directly participate in the restoration of homeostasis [24, 27], we sought to investigate the role of NPM1 in DNA DSB repair deficiencies in C9ALS/FTD focusing on the role of arginine-rich DPRs that are most frequently associated with nucleolar dysfunction [9, 10]. We found that a synthetic PR construct co-localizes with NPM1 in the nucleolus, but also the nucleoplasm and cytoplasm (Fig. 1). Inhibition of NPM1 by PR across cellular compartments could interfere with multiple cellular processes involving NPM1 including rRNA biogenesis, nucleolar liquid-liquid phase dynamics, nucleocytoplasmic transport, apoptotic signaling and DNA damage repair, thereby conferring toxicity.

While many of these processes have already been implicated in C9ALS/FTD, there is increasing interest in the role of genomic instability resulting from genotoxic DNA DSBs and/or reduced DNA DSB repair efficiencies as a common feature of neurodegenerative diseases [36, 37, 39, 74, 75]. Increased DNA DSB frequency with a concomitant activation of response and repair machinery has been observed in C9ALS/FTD clinical tissues, iPSC motor neurons, and cells expressing synthetic DPRs [52, 71, 72]. Assessments of C9ALS/FTD spinal cord tissues demonstrate enrichment of γH2AX [52, 72], poly(ADP-ribose) polymerase 1 (PARP-1) and P53 binding protein 1 (53BP1) [52]. Enrichment of γH2AX and phosphorylated P53 has also been observed in C9ALS motor neurons [71]. While there is general consensus that arginine-rich DPRs are neurotoxic, the degree of GA toxicity has been debated and its role in DNA damage is unclear. Lopez et al. found that GA overexpression did not increase DNA DSBs in control iPSC neurons, as determined by comet assay [71] while Walker et al. found that GA overexpression increased γH2AX levels both in vitro and in the rodent brain [73]. Recent findings also support a role for GA in aberrant ATM signaling and increased DNA DSB [76]. These discrepancies might be explained by the use of different cell types, number of dipeptide repeats used, and degree of overexpression. Moreover, these previous studies, have largely assessed levels of DNA repair proteins or aberrant DNA damage response signaling but did not evaluate the efficiency of specific repair pathways directly. Therefore, to better define the role of DPRs in DNA DSB repair pathways, we used a functional I-SceI DNA DSB repair assay to evaluate the efficiency of all major DNA DSB repair pathways in the presence of DPRs. We show that PR robustly reduced the efficiency of SSA and NHEJ, two pathways utilized by neurons. We also found that GA and GR significantly reduced NHEJ efficiency, while GA also significantly reduced SSA (Fig. 2). One limitation of these experiments is the utilization of immortalized U-2 OS cancer cell lines that may not accurately model neuronal DNA DSB repair. To address this limitation and confirm our findings in more disease-relevant model systems, we interrogated levels of RAD52 in patient-derived neurons and isogenic control lines. We found that genome editing reduces the accumulation of DNA DSBs and RAD52 hyperactivation. It should be noted, however, that this approach eliminates all potentially toxic HRE products including mutant C9ORF72 mRNA, antisense RNA and all five DPRs. Thus, we cannot attribute the reversal of activated pRAD52 and total RAD52 signaling in C9ALS/FTD iPSC neurons to the loss of DPRs alone. While we observed a high degree of variability in the expression of RAD52 across clinical samples, a statistically significant increase was found in C9ALS samples, as compared to unaffected controls or sALS samples (Fig. 6). Our observation that RAD52 is elevated in all brain regions, including those typically assumed to be unaffected, is not unexpected. Even the most prominent molecular features of C9ALS/FTD (e.g. DPRs and RNA foci) have a confounding relationship with clinicopathology. It is widely recognized that the presence or levels of DPRs are not predictive of the degree of neurodegeneration in a given region of the CNS. Apparently, resistance to toxicity varies across cell types and brain regions, for unknown reasons [77]. Our analysis of clinical brain tissues confirmed increased expression of both RAD52 and poly(GP) in all C9ALS/FTD samples (Additional file 13). The limited number and quality of the sample cohort used in this study is a potential weakness (Additional file 11).

In replicating cells, NPM1 is recruited to sites of DNA damage where it co-localizes with components of repair machinery including γH2AX, BRCA1 and ring finger 8 and 168 (RNF8/RNF168) [53, 78]. Moreover, NPM1 loss-of-function destabilizes the genome [79–82] while overexpression enhances DNA repair capacity and improves survival of cells exposed to ultra-violet radiation [83]. Conversely, reduced NPM1 function increases radiation sensitivity in vitro and stalls DNA DSB repair, suggesting NPM1-mediated repair may be rate limiting [53, 84]. Consistent with the protective role of NPM1 in C9ALS/FTD, Farg et al. found that overexpression of NPM1 reduced pro-apoptotic signaling in response to ectopic PR expression [52]. These studies, and those of others [35, 52, 74, 78, 85] implicate NPM1 in DNA damage repair, although its role in specific DNA DSB repair pathways has not been sufficiently addressed. Here we show that chemical induction of DNA DSBs in vitro results in the translocation of NPM1 from nucleoli to the nucleoplasm where it co-localizes with pRAD52, a specific marker of SSA (Fig. 4). Moreover, depletion of NPM1 significantly reduces the efficiency of NHEJ and SSA (Fig. 3), thereby implicating a ubiquitous role for NPM1 in both homology-directed and non-homologous repair pathways. Our data support the notion that DPRs impair DNA DSB through inhibition of NPM1’s role in DNA DSB repair (Additional file 14). To our knowledge, ours is the first study to link NPM1 to SSA. Future studies will be needed to fully disentangle the multifaceted role of NPM1 in C9ALS/FTD and its mechanistic role in SSA.

In addition to NPM1, several ALS-linked RNA binding proteins have direct roles in DNA DSB repair including valosin-containing protein (VCP), fused in sarcoma (FUS) and TAR DNA binding protein 43 (TDP-43) [70, 72, 86–92]. It is increasingly understood that the RNA processing functions of these proteins are capable of destabilizing co-transcriptional structures called R-loops, which are composed of the nascent RNA hybridized with template DNA and the non-template single-stranded DNA, thereby reducing the potential of persistent R-loops to result in DNA DSBs [73, 93, 94]. In addition to preventing DNA DSBs from occurring during transcription, these proteins may also facilitate transcription-associated homology-directed repair in post-mitotic neurons [41, 42, 92]. Interestingly, RAD52 plays a key role in RNA-coordinated DNA DSB repair by tethering complementary single strand DNA and RNA to facilitate annealing during homology-directed repair and has been linked to R-loop processing and transcription-associated repair [40, 42, 43, 95, 96]. Thus, nascent RNAs, RNA binding proteins, and RAD52 have key roles in maintaining the integrity of active neuronal genes, roles that are compromised by the presence of DPRs. Our findings support this model by 1) demonstrating that DPRs inhibit DSB repair efficiency, 2) indicating that NPM1 facilitates homology-directed DNA DSB repair and 3) identifying constitutively active RAD52 as a novel molecular phenotype in C9ALS/FTD. Future studies will be needed to fully elucidate the role of NPM1, FUS, TDP-43 and other ALS-linked RNA binding proteins in preventing R-loop associated DNA DSBs as well as their role in facilitating homology-directed DNA DSB repair.

## Conclusions

Here we show that C9ORF72 DPRs inhibit multiple DNA DSB repair pathways that are utilized by post-mitotic neurons to maintain genomic integrity throughout their extended lifetime. In addition to identifying aberrant homology-directed DNA DSB repair as a novel C9ORF72-related disease mechanism, we show that impairment of SSA is partially mediated through NPM1 inhibition. Lastly, these results support emerging evidence that RNA binding proteins like NPM1 and homology-directed repair machineries including RAD52 play critical roles in RNA-directed DNA DSB repair.

## Supplementary information

**Additional file 1.** Antibody applications and dilutions.

**Additional file 2 **Nucleolar localization of PR is dependent on NPM1 expression. A) Representative images of U-2 OS cells transfected with a plasmid expressing HA-PR (PR) and immunolabeled with anti-PR (red) and anti-nucleophosmin (NPM1) (green) antibodies. Localization of the nucleus DAPI (blue), NPM1 (green), and PR (red) were visualized by confocal microscopy. B) Nuclear PR mean fluorescence intensity is significantly reduced in NPM1 siRNA treated cells when compared to control siRNA treated cells. C) NPM1 knock down significantly reduces mRNA levels determined through real time PCR. Cytoplasmic PR levels are increased when co-expressed with GFP-NPM1-NLSΔ. D) Quantification of dot blot for PR in the cytoplasm relative to GAPDH. Significance calculated using student’s t-test, *n* = 3 biological replicates; error bars are SEM; *P < 0.05, ****p < 0.0001.

**Additional file 3 **Transection efficiency is not altered by DPR expression. U2-OS cells were co-transfected with a GFP expression plasmid (1 μg) and either pcDNA, PR, GR, or GA plasmids (1 μg). Using FACS, we quantified the number of GFP expressing cells and found no significant change in the number of GFP expressing cells between groups, indicating that DPRs do not alter the transfection efficiency of other plasmids. Three replicates for each experimental group; *n* > 50,000 cells/sample; error bars are SEM.

**Additional file 4.** Validation of NPM1 knockdown and PR overexpression. In parallel with the quantification of SSA and NHEJ (Fig. 3), a subset of cells were transfected with either a control (CTL) siRNA, a NPM1 specific siRNA, an empty control vector (vector) or a PR expression vector (PR). (A) To confirm NPM1 depletion, RNA was extracted from three biological replicates and analyzed by real-time RT-PCR using the relative quantification method where GAPDH served as the endogenous control. Cells transfected with the NPM1 siRNA has significantly lower levels of NPM1 mRNA (****p < 0.0001). (B) Also in parallel, proteins were isolated from two biological replicates and analyzed by western blot. Relative to the endogenous control (Actin), NPM1 levels were drastically reduced. (C) To confirm the overexpression of PR in cells transfected with the PR overexpression vector (PR) or an empty control vector (vector), the insoluble nuclear protein fraction was isolated and a slot-blot was performed, hybridized with the anti-PR antibody then visualized by chemiluminescence. A robust increase in PR expression was readily observed.

**Additional file 5 **PR overexpression and NPM1 depletion increase levels of the DNA double strand break marker γ-H2AX. A) Western blot analysis of U-2 OS cells co-transfected with the HA-PR plasmid and an NPM1 siRNA at 48 h. B) Western blot analysis of U-2 OS etoposide treated cells with or without NPM1 siRNA. C) Western blot used for A and B quantifications. **P < 0.005, ***P < 0.0005 relative to pcDNA3.1+ control; *n* = 3 biological replicates, one-way ANOVA followed by Tukey’s post-hoc test; error bars are SEM.

**Additional file 6.** Validation of DNA damage induction. A) DNA double strand break inducers validated through immunoblotting. B) Super resolution (STORM) microscopy reveals increased γH2AX (red) and phosphorylated RAD52 (green) immunofluorescence and co-localization (yellow) in the nucleus of U-2 OS cells treated with etoposide.

**Additional file 7 **Quantification of DNA damage markers in patient derived motor neurons. Western blot analysis of total protein lysates from motor neurons derived from two iPSC lines from unaffected controls (CTL-1, CTL-2) and two lines from C9ALS patients (C9ALS-1, C9ALS-2) resolved by electrophoresis and immunolabeled with antibodies against a marker of DNA damage foci, γH2AX (A), a marker of non-homologous end joining recombination repair, Ku70 (B) or single strand annealing, RAD52 (C). Statistical significance was assessed by one-way ANOVA and post-hoc test between each group; *n* = 2 biological replicates; error bars are SEM; *p < 0.05, **p < 0.005, ***p < 0.0005, ****p < 0.0001. D) DNA methylation analysis of CpG dinucleotides at the C9ORF72 promoter. Reduced DNA DSB markers for C9ALS-2 neurons is associated with increased CpG methylation at the C9ORF72 promoter.

**Additional file 8.** Genome editing eliminates the C9ORF72 hexanucleotide repeat expansion. A) Schematic of editing strategy: adeno-associated viral (AAV) transduction of iPSCs derived from a C9ALS/FTD patient, titration, clonal selection and isolation of genomic DNA for downstream analysis. Location of guide RNAs and PCR primers spanning the HRE. Expected amplicon sizes for the wild type (WT), expanded and edited alleles are indicated. Electrophoresis of PCR amplicons from three iPSC clones C9ALS-1.4 (HRE+/wt-), C9ALS-1.8 (HRE+/wt+), C9ALS-1.11 (HRE−/wt-) using C9 flanking primers (top) or template input control primers (bottom). Sanger sequencing of PCR amplicons from clone C9ALS-1.11 (HRE−/wt-) confirms loss of sequence homology to the reference genome between gRNAs. Schematic of repeat primed PCR amplification of the HRE. Fragment analysis of amplicons for three clones confirms the loss of the HRE in clone C9ALS-1.11. B) Electropherogram output from fragment analysis indicating intensity (Y-axis) and size (X-axis) of amplicons produced by C9ORF72 repeat-primed PCR. Source of template genomic DNA corresponding to each iPSC cell line used in the study is indicated in upper-right corner. Prototypical saw-tooth pattern is evident in three patient derived cell lines with the hexanucleotide expansion (C9ALS-1, C9ALS-4, C9ALS-5) and lack of PCR products in gnomically edited cell lines (iso). analysis of amplicons for all 6 cell lines clones the loss of the HRE in all isogenic clones. C) Representative images of motor neurons from six iPSC lines stained for DAPI (blue) and immunolabeled with antibodies against Tuj1 (green) and PR (red).

**Additional file 9.** Expression of neuronal markers in iPSC motor neuron cultures. Representative images of neuronal cultures stained DAPI (blue) and antibodies against the neuronal nuclear envelope marker protein NeuN (top panels) and the motor-neuron specific marker ISL-1 (bottom panels).

**Additional file 10 **Quantification of DNA damage foci in C9ORF72 iPSC neurons. A) Representative images of iPSC neuronal cultures immuno-stained with γH2AX (green), Tuj-1 (red) and DAPI (blue). B-D) Quantification of γH2AX mean fluorescence normalized to isogenic line; *n* = 2, 5 fields; error bars are SEM; ****P < 0.0001, as determined by unpaired student’s t test.

**Additional file 11.** Target ALS Human Postmortem Brain Tissue Cohort. Target ALS Human Postmortem Brain Tissue Cohort. All diagnoses and identifiers were the same for the three brain regions provided (occipital cortex, cerebellum, and motor cortex).

**Additional file 12 **Quantification of phosphorylated RAD52 and 53BP1 in human brain samples. Quantification of pRAD52 (A,B) and 53BP1 (C,D) as assessed by western analysis of protein lysates from unaffected controls (CTL), C9ORF72 related ALS (C9ALS) and sporadic ALS (sALS); three different brain regions: Occipital cortex (OC) Cerebellum (CB) and Motor cortex (M1). Comparisons between diagnosis groups were performed by mixed effect analyses utilizing data from all three brain regions and accounting for both the between-region differences and within-person correlation; *n* = 6 per diagnosis group, 3 measurements per person – one from each region. E) One-way ANOVA Tukey’s post-hoc comparison between diagnosis groups for each brain region.

**Additional file 13 **Quantification of poly(GP) in human brain samples. A) Quantification of poly(GP) in protein lysates from unaffected controls (CTL), C9ORF72 related ALS (C9ALS) and sporadic ALS (sALS) in three different brain regions: Occipital cortex (OC), Cerebellum (CB) and Motor cortex (M1). Poly(GP) levels were measured using a Meso Scale Discovery – based immunoassay. Each sample was measured in duplicate and the mean values are represented. Comparisons between groups were performed by one-way ANOVA for each brain region. *N* = 6 per diagnosis group, 3 measurements per person – one from each region. **** = P < 0.0001 B) One-way ANOVA Tukey’s post-hoc comparison between diagnosis groups for each brain region.

**Additional file 14.** Model of DPR-mediated inhibition of DNA DSB repair. In nucleoli, PR binds and stabilizes NPM1 pentamers by binding to the acidic loop of NPM1 in a similar way that endogenous arginine-rich proteins like ARF bind and stabilize NPM1. Since NPM1 facilitates DNA DSB repair in the nucleoplasm as a monomer, we hypothesize that PR inhibits DNA DSB repair, in part, by preventing the translocation of NPM1 from the nucleolus to the nucleoplasm.

## Data Availability

The datasets supporting the conclusions of this article are included within the article and its additional files.

## Abbreviations

53BP1: P53 binding protein 1
AAV: Adeno-associated virus
ACTB: Beta-actin
ALS: Amyotrophic lateral sclerosis
BRCA2: Breast cancer 2
C9ALS: C9ORF72-related ALS
C9ORF72: Chromosome 9 open reading frame 72
CB: Cerebellum
CE: Capillary electrophoresis
DPR: Dipeptide repeat protein
DSB: Double strand break
FACS: Fluorescence activated cell sorting
FTD: Frontotemporal Dementia
FUS: Fused in sarcoma
GA: Glycine-alanine
GP: Glycine-proline
GR: Glycine-arginine
gRNAs: Guide RNAs
HRE: Hexanucleotide repeat expansion
ICC: Immunocytochemistry
iMN: Induced pluripotent stem cell-derived motor neuron
iPSC: Induced pluripotent stem cell
M1: Motor cortex
MMEJ: Microhomology-mediated end joining
NCL: Nucleolin
NHEJ: Non-homologous end joining
NPM1: Nucleophosmin
OC: Occipital cortex
PARP-1: Poly(ADP-ribose) polymerase 1
PR: Proline-arginine
pRAD52: Phosphorylated RAD52
RNF8/RNF168: Ring finger 8 and 168
rRNA: Ribosomal RNA
sALS: Sporadic ALS
siRNA: Small inhibitory RNA
SSA: Single strand annealing
STORM: Stochastic optical reconstruction microscopy
TDP-43: TAR DNA binding protein 43
tRAD52: Total RAD52
VCP: Valosin-containing protein
WT: Wild-type
