# Supplementary material for: Dipeptide repeat proteins inhibit homology-directed DNA double strand break repair in C9ORF72 ALS/FTD
Source: Mol Neurodegener. 2020 Feb 24;15:13. doi: 10.1186/s13024-020-00365-9 (PMC7041170; doi:10.1186/s13024-020-00365-9)
Supplement: Supplementary file 1 — Additional file 1. Antibody applications and dilutions. [file 13024_2020_365_MOESM1_ESM.pdf]

Additional file 1. Antibody applications and dilutions

Antibody	Supplier	Catalog #	Dilution	Method
anti-HA	Abcam	ab130275	1:200	IF
anti-RAD52	ThermoFisher	PA5-65036	1:250	IF
anti-pRAD52	ThermoFisher	PA5-38702	1:100,1:500	IF,WB
anti-bActin	Sigma	a1978	1:2000	WB
anti-MAP2	Abcam	ab5392	1:500	IF
anti-Tuj1	Abcam	ab78078	1:500,1:1000	IF,WB
anti-yH2AX	Abcam	ab11174	1:1000	WB
anti-yH2AX	ThermoFisher	MA5-27753	1:500	IF
anti-PR	ProteinTech	23979-1-AP	1:250,1:500	IF,WB
anti-RAD52	Abcam	ab124971	1:1000	WB
