# Supplementary figures and images for: Dipeptide repeat proteins inhibit homology-directed DNA double strand break repair in C9ORF72 ALS/FTD
Source: Mol Neurodegener. 2020 Feb 24;15:13. doi: 10.1186/s13024-020-00365-9 (PMC7041170; doi:10.1186/s13024-020-00365-9)

**A**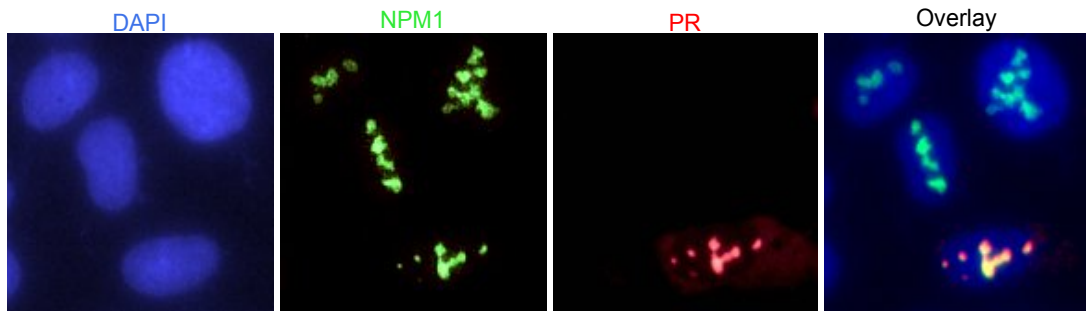**B**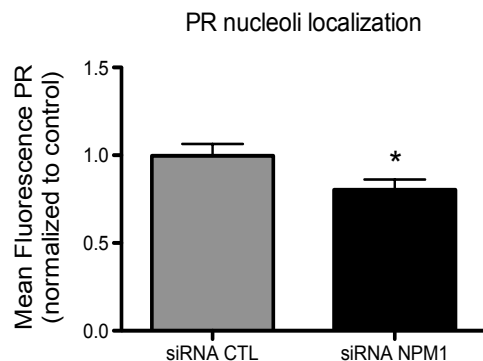**C**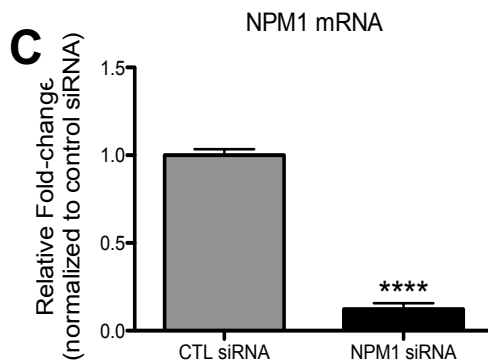**D**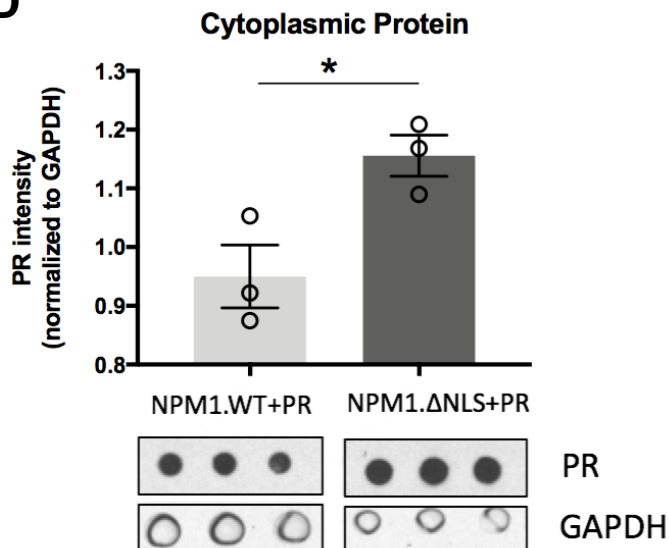

Supplement: Supplementary file 2 — Additional file 2 Nucleolar localization of PR is dependent on NPM1 expression. A) Representative images of U-2 OS cells transfected with a plasmid expressing HA-PR (PR) and immunolabeled with anti-PR (red) and anti-nucleophosmin (NPM1) (green) antibodies. Localization of the nucleus DAPI (blue), NPM1 (green), and PR (red) were visualized by confocal microscopy. B) Nuclear PR mean fluorescence intensity is significantly reduced in NPM1 siRNA treated cells when compared to control siRNA treated cells. C) NPM1 knock down significantly reduces mRNA levels determined through real time PCR. Cytoplasmic PR levels are increased when co-expressed with GFP-NPM1-NLSΔ. D) Quantification of dot blot for PR in the cytoplasm relative to GAPDH. Significance calculated using student’s t-test, n = 3 biological replicates; error bars are SEM; *P < 0.05, ****p < 0.0001. [file 13024_2020_365_MOESM2_ESM.pdf]

## Transfection Efficiency

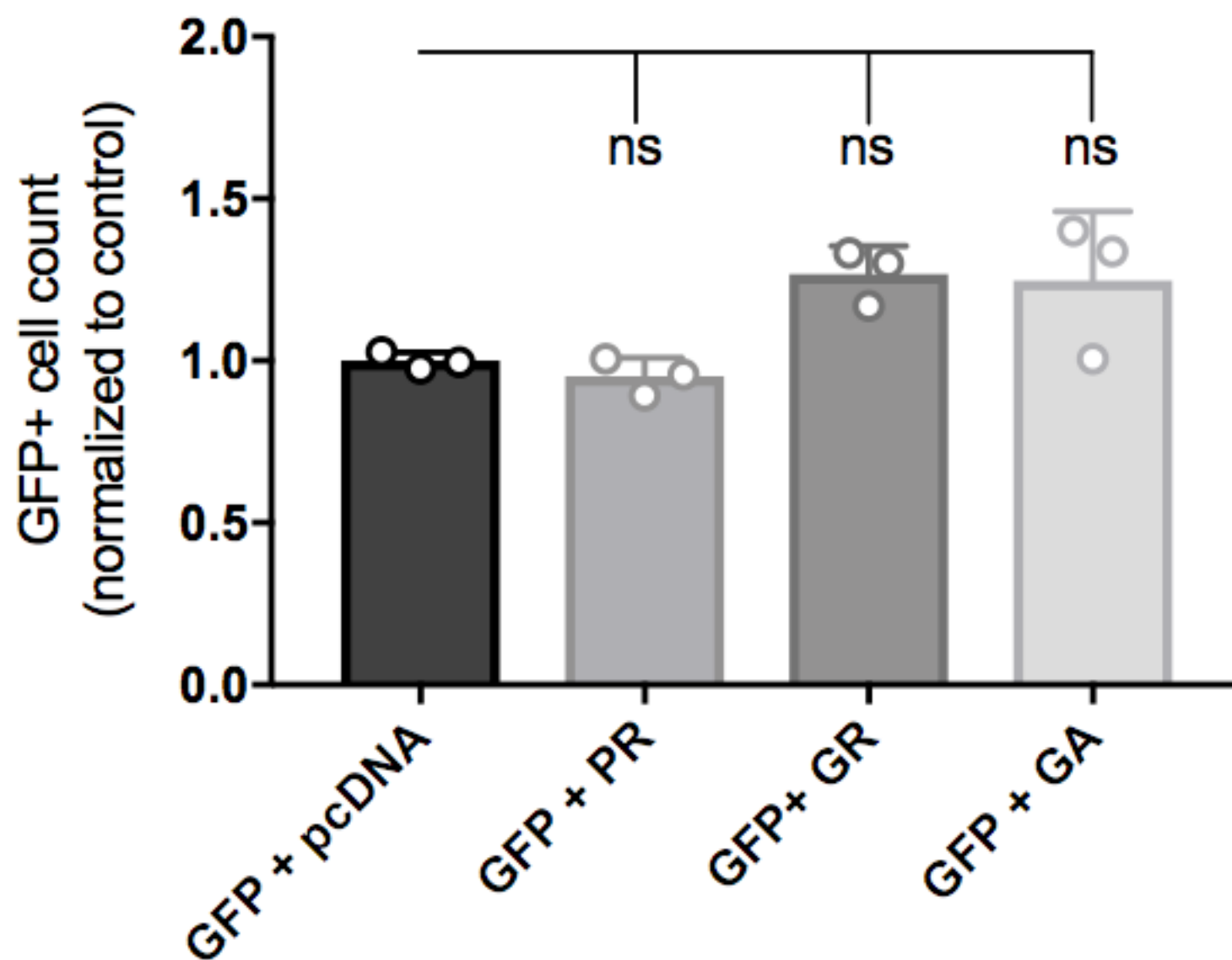

Supplement: Supplementary file 3 — Additional file 3 Transection efficiency is not altered by DPR expression. U2-OS cells were co-transfected with a GFP expression plasmid (1 μg) and either pcDNA, PR, GR, or GA plasmids (1 μg). Using FACS, we quantified the number of GFP expressing cells and found no significant change in the number of GFP expressing cells between groups, indicating that DPRs do not alter the transfection efficiency of other plasmids. Three replicates for each experimental group; n > 50,000 cells/sample; error bars are SEM. [file 13024_2020_365_MOESM3_ESM.pdf]

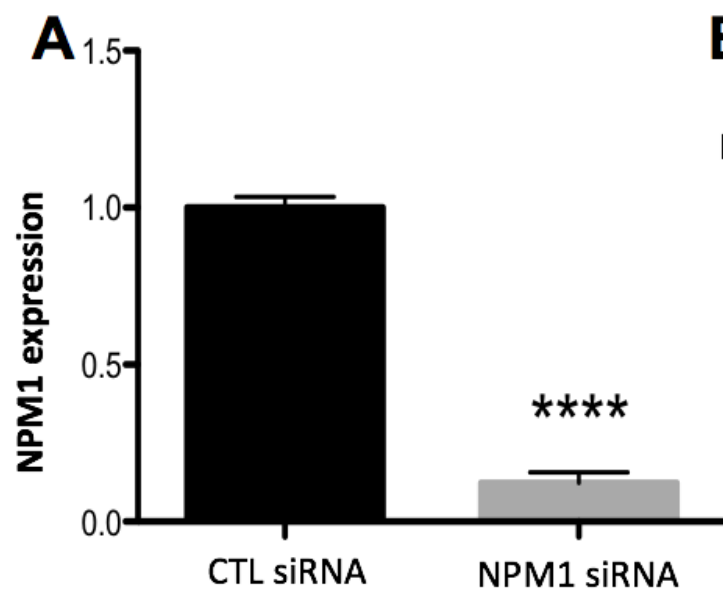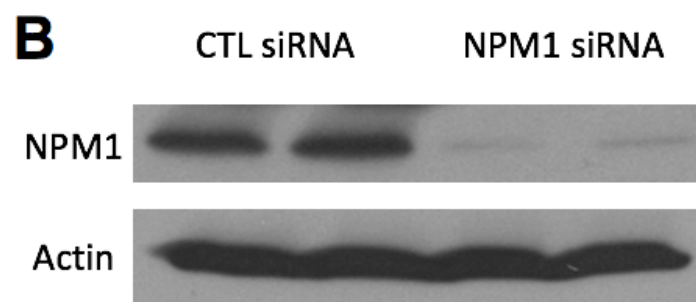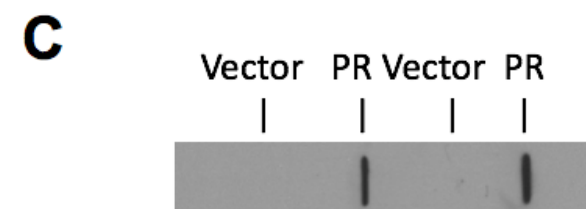

Supplement: Supplementary file 4 — Additional file 4. Validation of NPM1 knockdown and PR overexpression. In parallel with the quantification of SSA and NHEJ (Fig. 3), a subset of cells were transfected with either a control (CTL) siRNA, a NPM1 specific siRNA, an empty control vector (vector) or a PR expression vector (PR). (A) To confirm NPM1 depletion, RNA was extracted from three biological replicates and analyzed by real-time RT-PCR using the relative quantification method where GAPDH served as the endogenous control. Cells transfected with the NPM1 siRNA has significantly lower levels of NPM1 mRNA (****p < 0.0001). (B) Also in parallel, proteins were isolated from two biological replicates and analyzed by western blot. Relative to the endogenous control (Actin), NPM1 levels were drastically reduced. (C) To confirm the overexpression of PR in cells transfected with the PR overexpression vector (PR) or an empty control vector (vector), the insoluble nuclear protein fraction was isolated and a slot-blot was performed, hybridized with the anti-PR antibody then visualized by chemiluminescence. A robust increase in PR expression was readily observed. [file 13024_2020_365_MOESM4_ESM.pdf]

**A**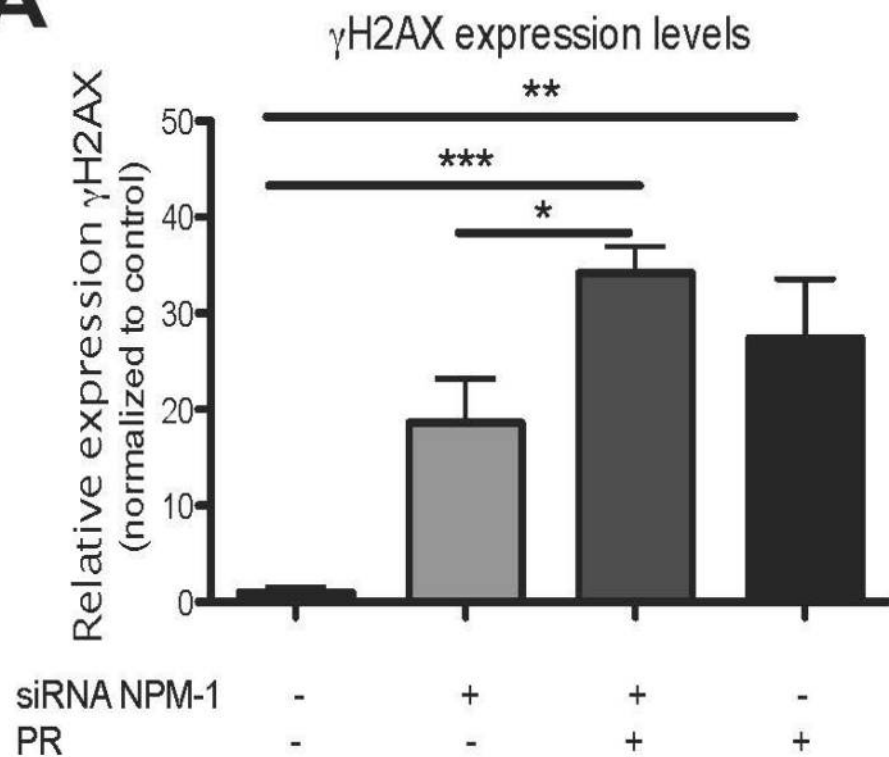**B**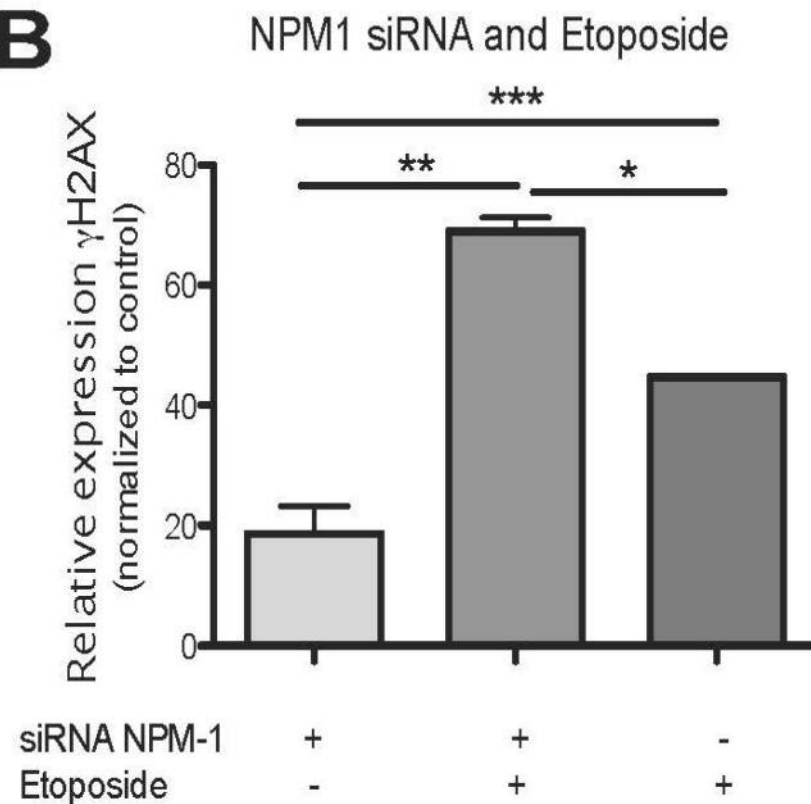**C**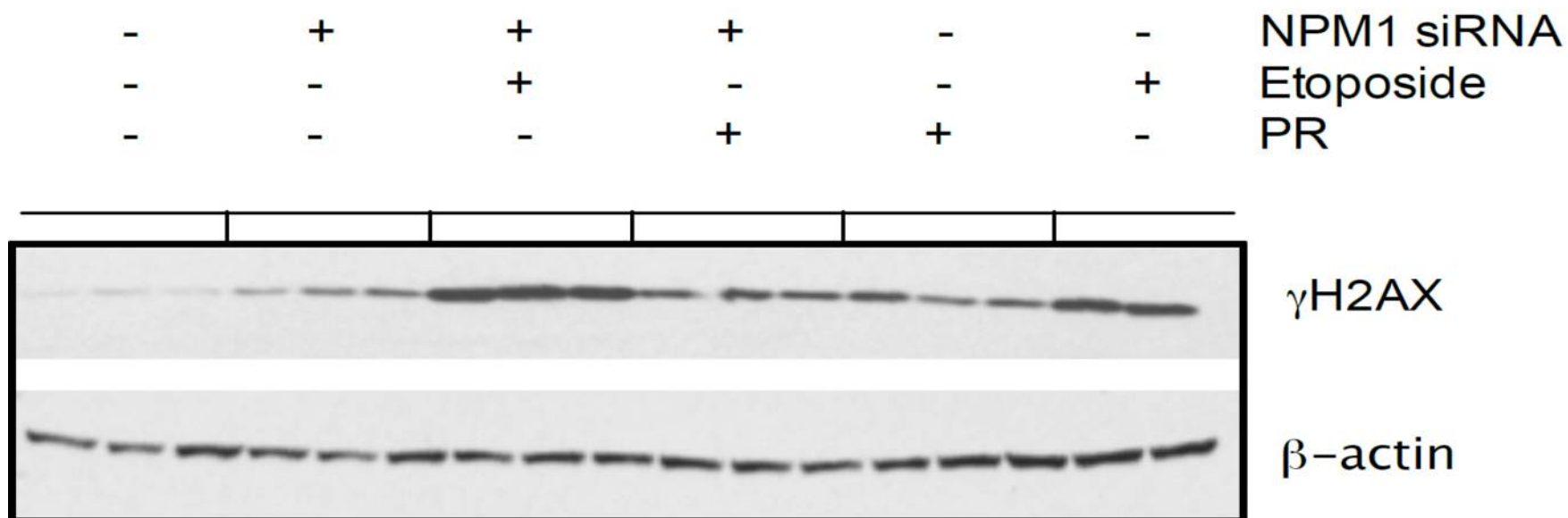

Supplement: Supplementary file 5 — Additional file 5 PR overexpression and NPM1 depletion increase levels of the DNA double strand break marker γ-H2AX. A) Western blot analysis of U-2 OS cells co-transfected with the HA-PR plasmid and an NPM1 siRNA at 48 h. B) Western blot analysis of U-2 OS etoposide treated cells with or without NPM1 siRNA. C) Western blot used for A and B quantifications. **P < 0.005, ***P < 0.0005 relative to pcDNA3.1+ control; n = 3 biological replicates, one-way ANOVA followed by Tukey’s post-hoc test; error bars are SEM. [file 13024_2020_365_MOESM5_ESM.pdf]

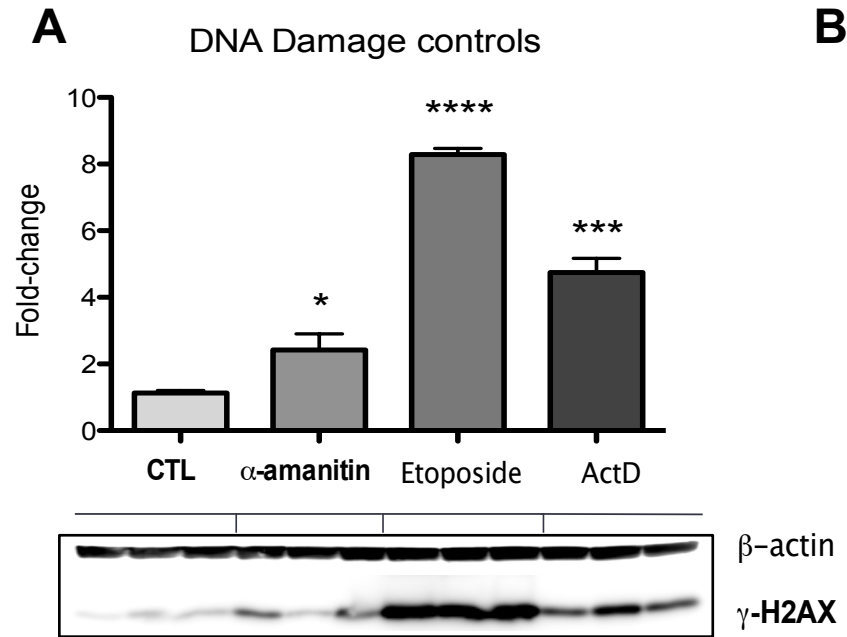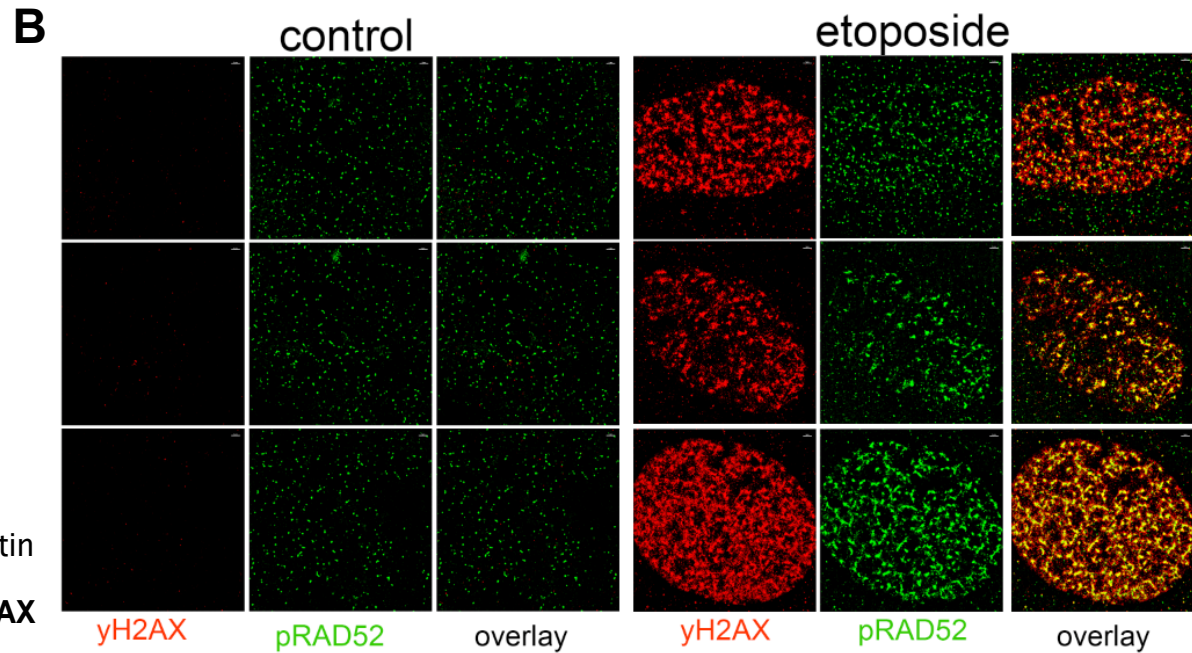

Supplement: Supplementary file 6 — Additional file 6. Validation of DNA damage induction. A) DNA double strand break inducers validated through immunoblotting. B) Super resolution (STORM) microscopy reveals increased γH2AX (red) and phosphorylated RAD52 (green) immunofluorescence and co-localization (yellow) in the nucleus of U-2 OS cells treated with etoposide. [file 13024_2020_365_MOESM6_ESM.pdf]

DAPI

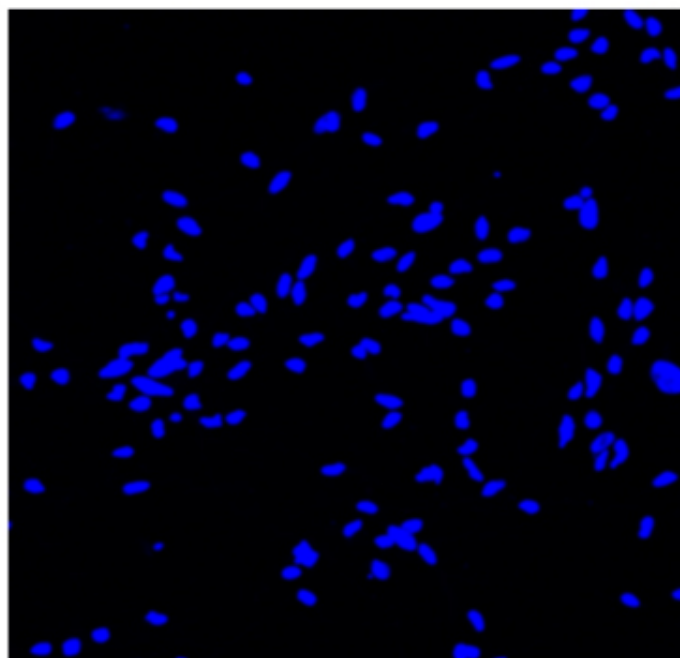

NeuN

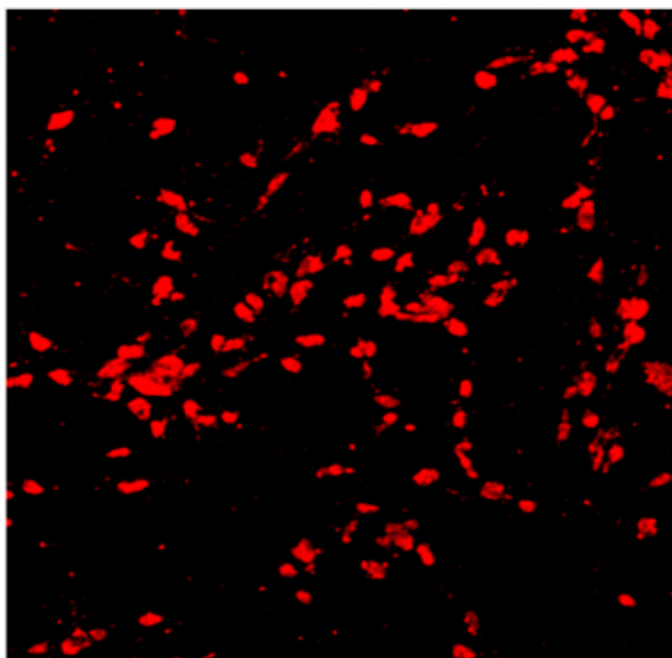

Merge

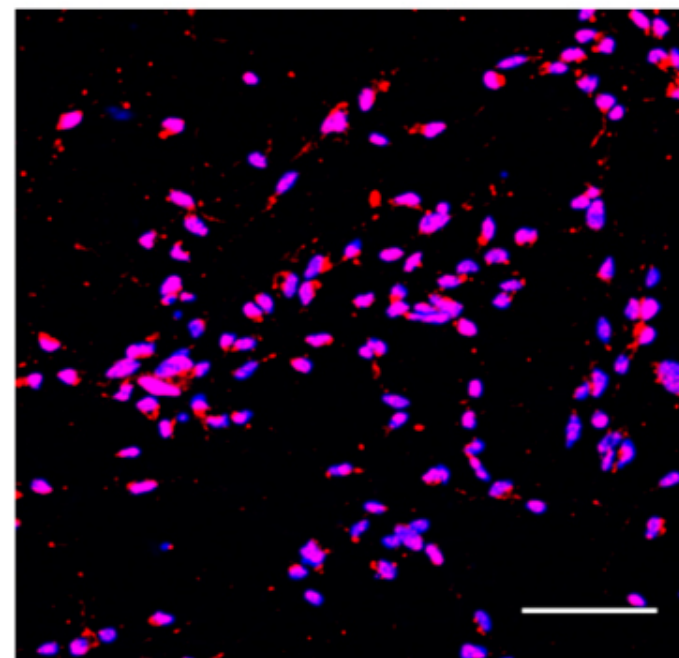

DAPI

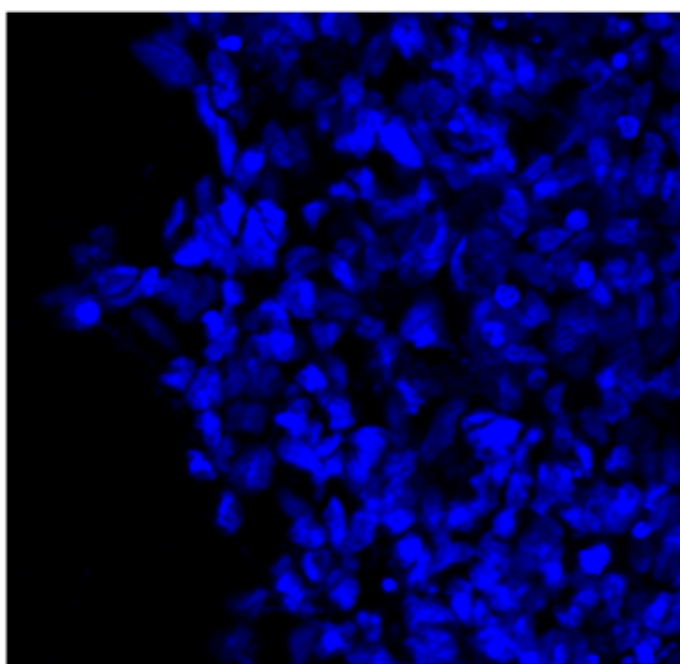

ISL1

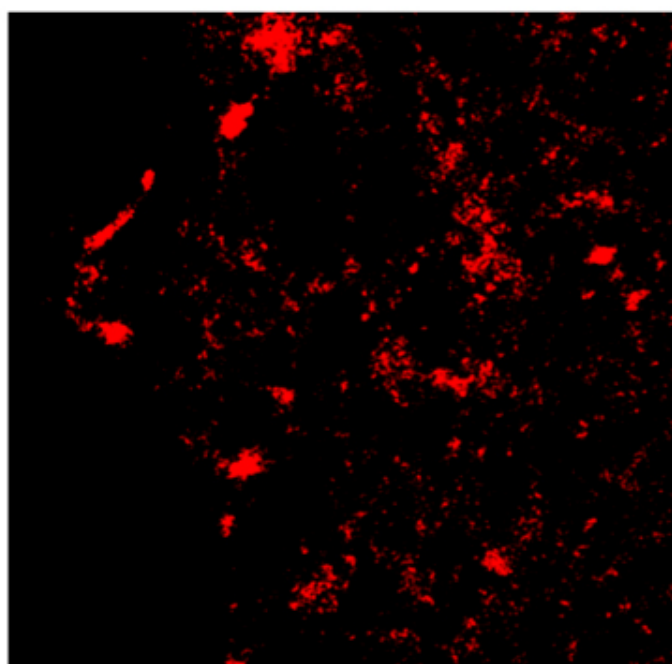

Merge

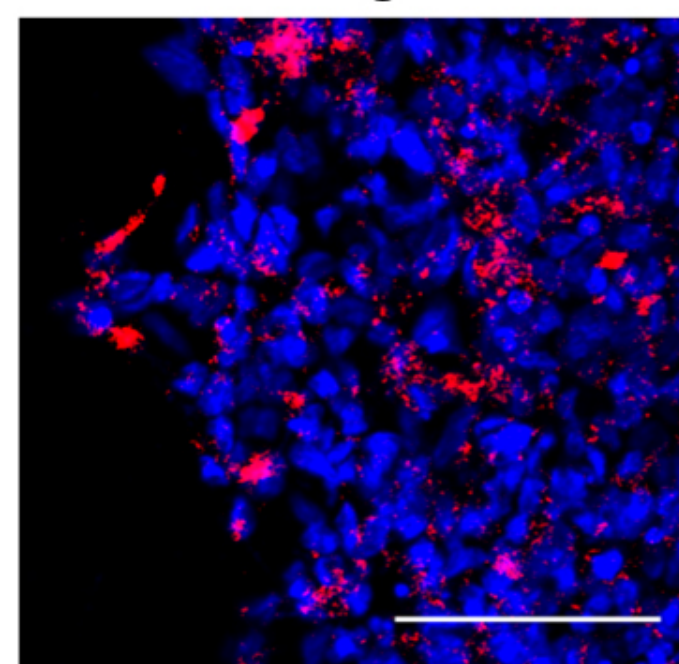

Supplement: Supplementary file 9 — Additional file 9. Expression of neuronal markers in iPSC motor neuron cultures. Representative images of neuronal cultures stained DAPI (blue) and antibodies against the neuronal nuclear envelope marker protein NeuN (top panels) and the motor-neuron specific marker ISL-1 (bottom panels). [file 13024_2020_365_MOESM9_ESM.pdf]

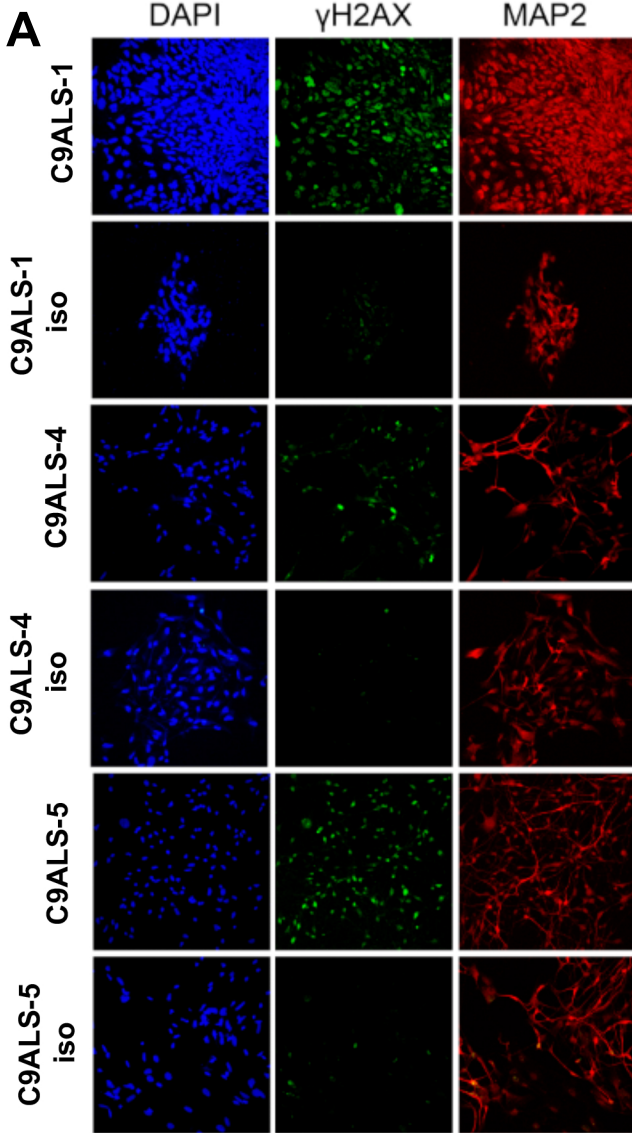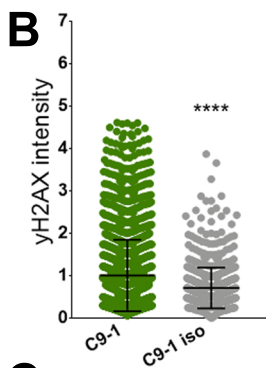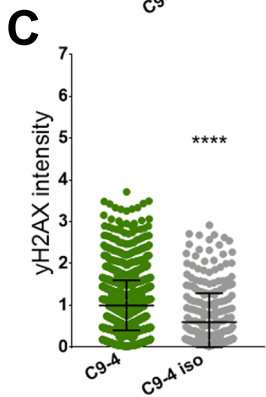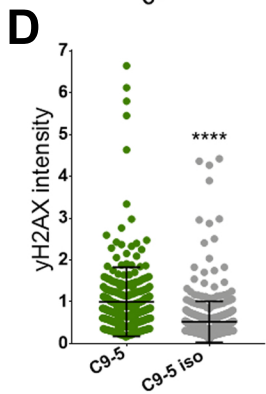

Supplement: Supplementary file 10 — Additional file 10 Quantification of DNA damage foci in C9ORF72 iPSC neurons. A) Representative images of iPSC neuronal cultures immuno-stained with γH2AX (green), Tuj-1 (red) and DAPI (blue). B-D) Quantification of γH2AX mean fluorescence normalized to isogenic line; n = 2, 5 fields; error bars are SEM; ****P < 0.0001, as determined by unpaired student’s t test. [file 13024_2020_365_MOESM10_ESM.pdf]

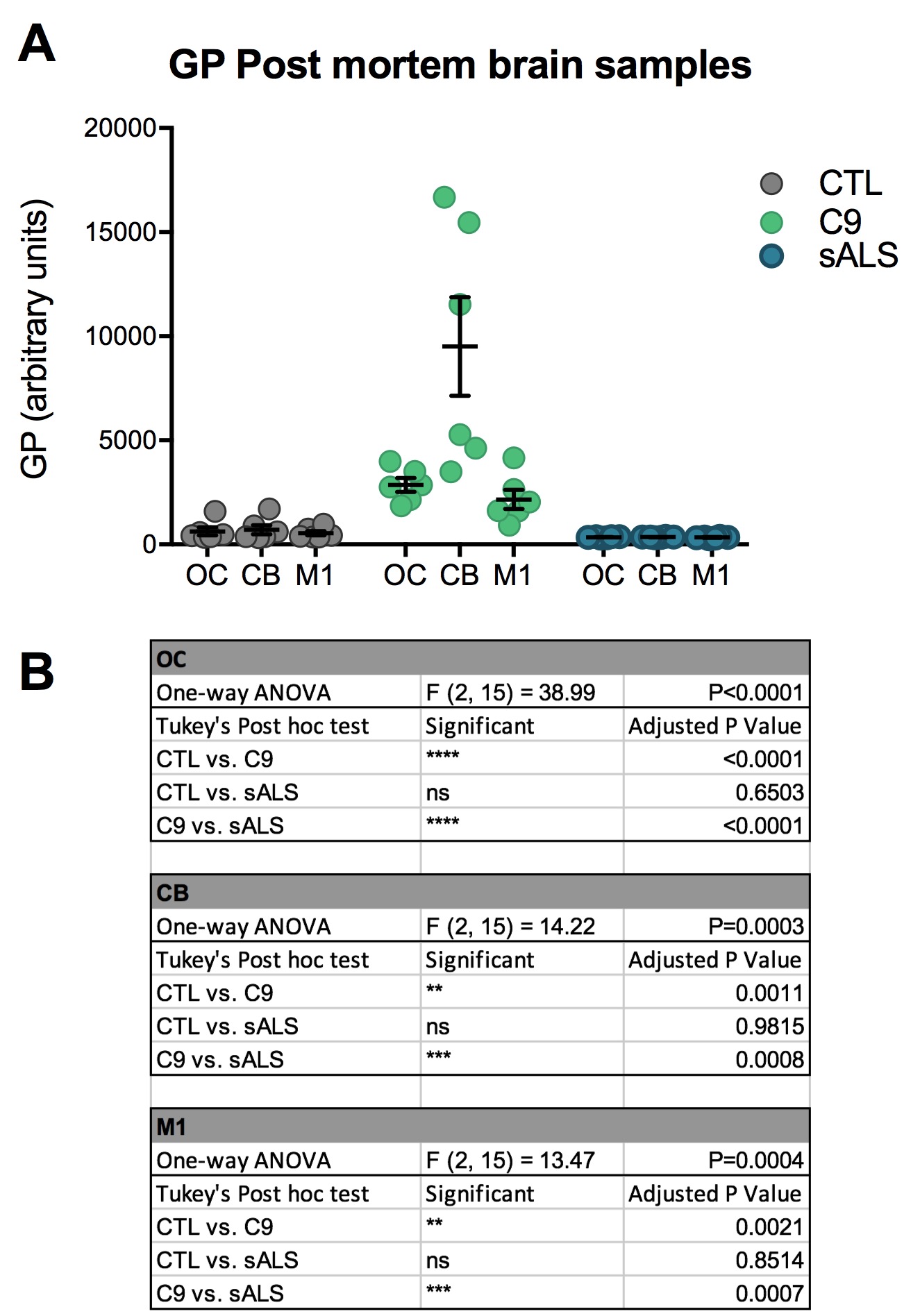

Supplement: Supplementary file 13 — Additional file 13 Quantification of poly(GP) in human brain samples. A) Quantification of poly(GP) in protein lysates from unaffected controls (CTL), C9ORF72 related ALS (C9ALS) and sporadic ALS (sALS) in three different brain regions: Occipital cortex (OC), Cerebellum (CB) and Motor cortex (M1). Poly(GP) levels were measured using a Meso Scale Discovery – based immunoassay. Each sample was measured in duplicate and the mean values are represented. Comparisons between groups were performed by one-way ANOVA for each brain region. N = 6 per diagnosis group, 3 measurements per person – one from each region. **** = P < 0.0001 B) One-way ANOVA Tukey’s post-hoc comparison between diagnosis groups for each brain region. [file 13024_2020_365_MOESM13_ESM.jpg]

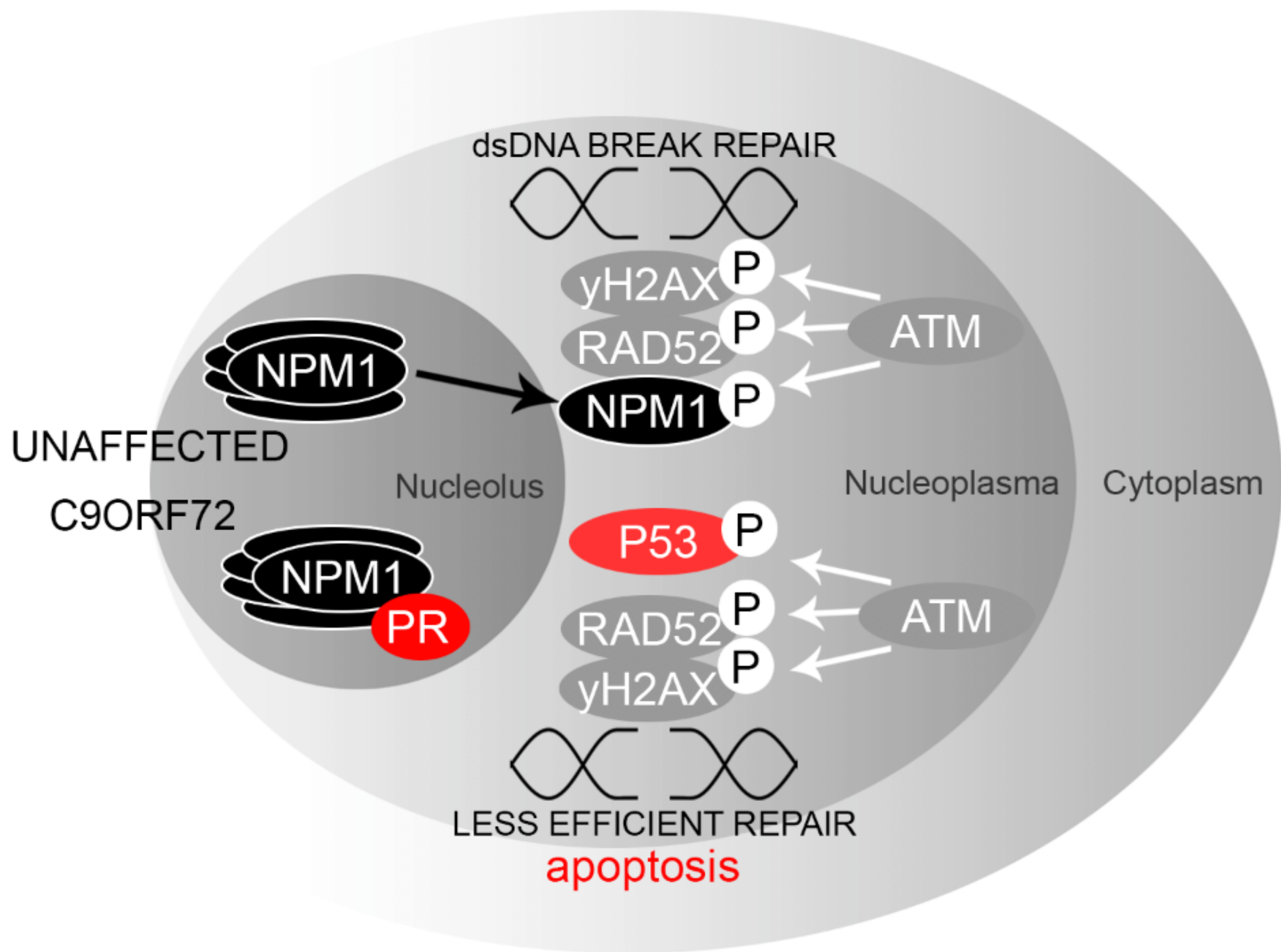

Supplement: Supplementary file 14 — Additional file 14. Model of DPR-mediated inhibition of DNA DSB repair. In nucleoli, PR binds and stabilizes NPM1 pentamers by binding to the acidic loop of NPM1 in a similar way that endogenous arginine-rich proteins like ARF bind and stabilize NPM1. Since NPM1 facilitates DNA DSB repair in the nucleoplasm as a monomer, we hypothesize that PR inhibits DNA DSB repair, in part, by preventing the translocation of NPM1 from the nucleolus to the nucleoplasm. [file 13024_2020_365_MOESM14_ESM.pdf]
