# Supplementary material for: Dipeptide repeat proteins inhibit homology-directed DNA double strand break repair in C9ORF72 ALS/FTD
Source: Mol Neurodegener. 2020 Feb 24;15:13. doi: 10.1186/s13024-020-00365-9 (PMC7041170; doi:10.1186/s13024-020-00365-9)
Supplement: Supplementary file 7 — Additional file 7 Quantification of DNA damage markers in patient derived motor neurons. Western blot analysis of total protein lysates from motor neurons derived from two iPSC lines from unaffected controls (CTL-1, CTL-2) and two lines from C9ALS patients (C9ALS-1, C9ALS-2) resolved by electrophoresis and immunolabeled with antibodies against a marker of DNA damage foci, γH2AX (A), a marker of non-homologous end joining recombination repair, Ku70 (B) or single strand annealing, RAD52 (C). Statistical significance was assessed by one-way ANOVA and post-hoc test between each group; n = 2 biological replicates; error bars are SEM; *p < 0.05, **p < 0.005, ***p < 0.0005, ****p < 0.0001. D) DNA methylation analysis of CpG dinucleotides at the C9ORF72 promoter. Reduced DNA DSB markers for C9ALS-2 neurons is associated with increased CpG methylation at the C9ORF72 promoter. [file 13024_2020_365_MOESM7_ESM.pdf]

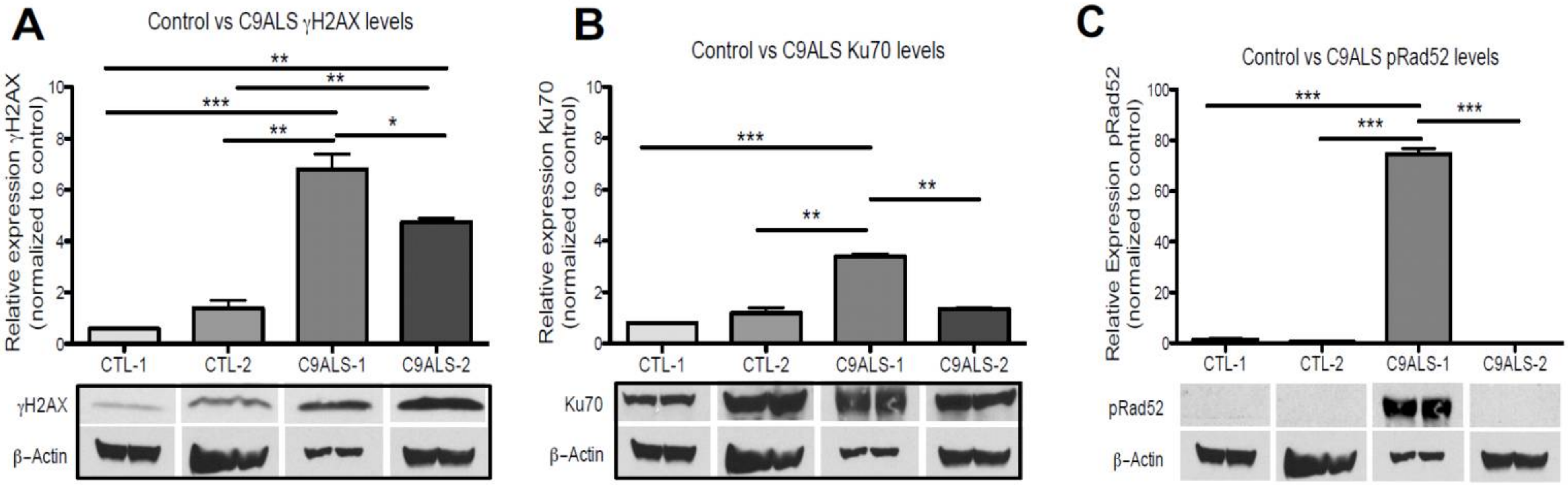

D

Human C9orf72 Methylation Analysis - Results in % Methylation											
		ADS3232-FS1								Overall Region	
From ATG		-6801	-6791	-6782	-6772	-6766	-6745	-6736	-6728	-6801 to -6728	
From TSS		-55	-45	-36	-26	-20	2	11	19	-55 to +19	
GRCh37/hg19, Chr9		27573921	27573911	27573902	27573892	27573886	27573865	27573856	27573848	chr9:27573921-27573848	
Customer ID	EpigenDx ID	CpG#-139	CpG#-138	CpG#-137	CpG#-136	CpG#-135	CpG#-134	CpG#-133	CpG#-132	Mean	St Dev
C9ALS-1_primary	B118882	34.7	41.7	8.3	7.4	51.4	10.4	8.1	4.2	20.8	18.7
C9ALS-1_iPSC	B118883	1.4	6.3	1.5	2.1	2.8	1.9	2.5	1.8	2.5	1.6
C9ALS-1_NPC	B118885	9.7	10.5	7.1	7.2	9.2	14.9	13.2	15.2	10.9	3.2
C9ALS-1_iMNs*	B118886	18.5	20.5	14.7	12.3	18.7	16.7	20.8	20.3	17.8	3.0
C9ALS-2_primary	B118878	1.8	2.4	0.0	0.0	0.0	0.0	0.0	0.0	0.5	1.0
C9ALS-2_iPSC	B118879	16.7	16.9	13.4	7.9	21.3	14.5	21.3	20.6	16.6	4.6
C9ALS-2_NPC	B118880	20.8	26.2	19.3	16.9	27.4	26.1	27.0	31.7	24.4	4.9
C9ALS-2_iMNs*	B118881	55.9	66.0	59.4	50.2	60.1	52.9	57.1	57.9	57.4	4.8
Methylation controls	Low	1.3	3.6	1.2	1.4	1.7	0.0	0.0	2.2	1.4	1.2
	Med	67.6	79.9	68.1	56.6	74.2	67.7	70.1	67.3	68.9	6.6
	High	87.9	98.2	91.7	76.8	98.2	74.3	84.1	88.6	87.5	8.8
