# Supplementary material for: Dipeptide repeat proteins inhibit homology-directed DNA double strand break repair in C9ORF72 ALS/FTD
Source: Mol Neurodegener. 2020 Feb 24;15:13. doi: 10.1186/s13024-020-00365-9 (PMC7041170; doi:10.1186/s13024-020-00365-9)
Supplement: Supplementary file 11 — Additional file 11. Target ALS Human Postmortem Brain Tissue Cohort. Target ALS Human Postmortem Brain Tissue Cohort. All diagnoses and identifiers were the same for the three brain regions provided (occipital cortex, cerebellum, and motor cortex). [file 13024_2020_365_MOESM11_ESM.pdf]

Additional file 11. Target ALS Human Postmortem Brain Tissue Cohort. All diagnosis and identifiers were the same for the three brain regions provided (occipital cortex, cerebellum, and motor cortex).

Site	Diagnosis	Sample Name	Identifier
Georgetown Brain Bank	Control	CTL_1	13-1
	Control	CTL_2	13-3
	Control	CTL_3	13-4
	Control	CTL_4	15-8
	Control	CTL_5	15-9
	Control	CTL_6	15-13
University of California San Diego	C9+ ALS	C9_ALS_1	14
	C9+ ALS	C9_ALS_2	91
	C9+ ALS	C9_ALS_3	98
	Sporadic ALS	sALS_1	27
	Sporadic ALS	sALS_2	30
	Sporadic ALS	sALS_3	32
Barrow Neurological Institute	C9+ ALS FTD+	C9_ALS_4	14-01
	C9+ ALS	C9_ALS_5	14-04
	C9+ ALS	C9_ALS_6	15-09
	Sporadic ALS	sALS_4	14-05
	Sporadic ALS	sALS_5	15-07
	Sporadic ALS	sALS_6	15-08
