# Supplementary material for: Dipeptide repeat proteins inhibit homology-directed DNA double strand break repair in C9ORF72 ALS/FTD
Source: Mol Neurodegener. 2020 Feb 24;15:13. doi: 10.1186/s13024-020-00365-9 (PMC7041170; doi:10.1186/s13024-020-00365-9)
Supplement: Supplementary file 12 — Additional file 12 Quantification of phosphorylated RAD52 and 53BP1 in human brain samples. Quantification of pRAD52 (A,B) and 53BP1 (C,D) as assessed by western analysis of protein lysates from unaffected controls (CTL), C9ORF72 related ALS (C9ALS) and sporadic ALS (sALS); three different brain regions: Occipital cortex (OC) Cerebellum (CB) and Motor cortex (M1). Comparisons between diagnosis groups were performed by mixed effect analyses utilizing data from all three brain regions and accounting for both the between-region differences and within-person correlation; n = 6 per diagnosis group, 3 measurements per person – one from each region. E) One-way ANOVA Tukey’s post-hoc comparison between diagnosis groups for each brain region. [file 13024_2020_365_MOESM12_ESM.pdf]

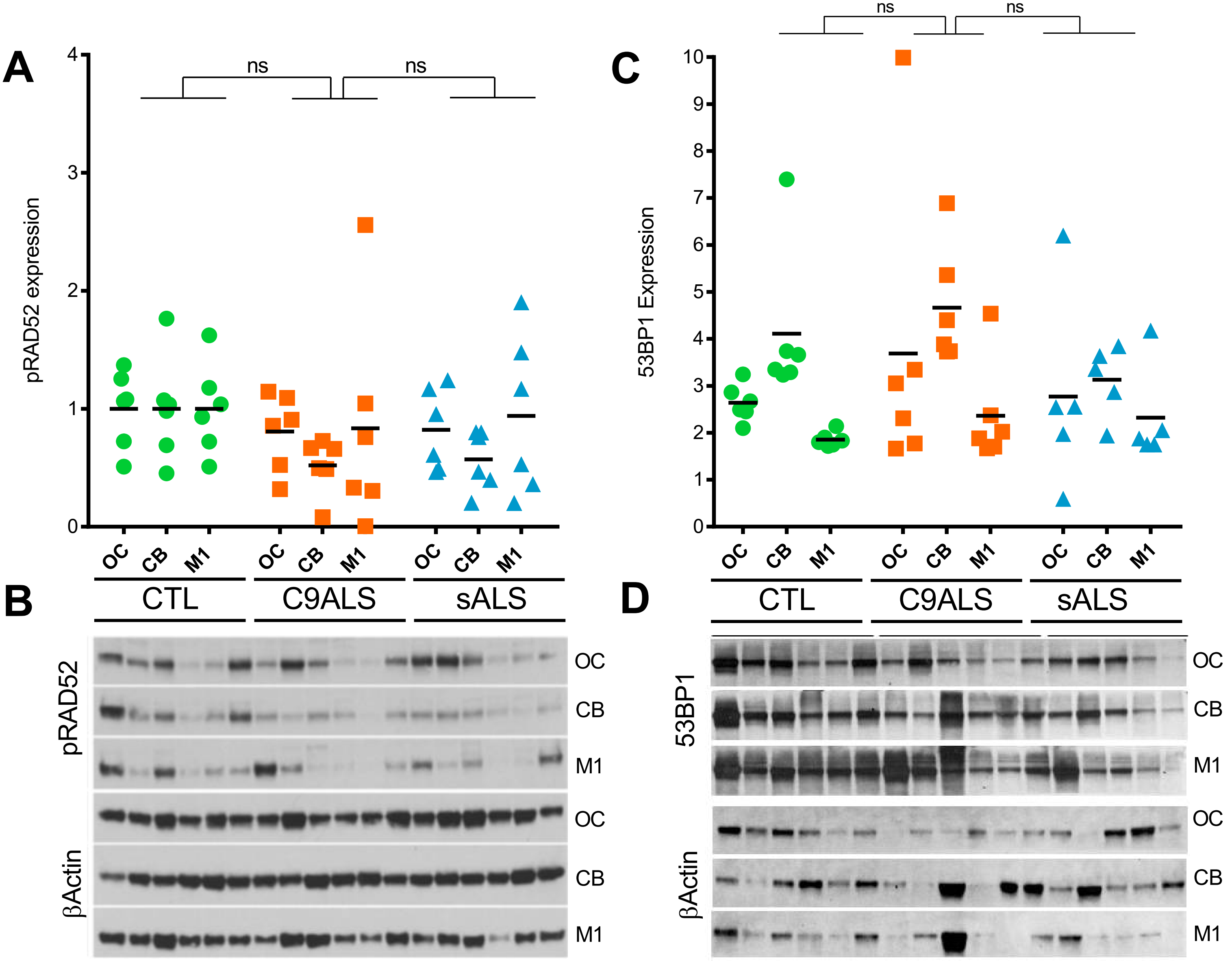

E

Tukey's Multiple Comparisons Test						
	RAD52		pRAD52		53BP1	
	Significant	P value	Significant	P value	Significant	P value
**Occipital Cortex**						
CTL vs C9ALS	**	0.0023	ns	0.5826	ns	0.8191
CTL vs sALS	ns	0.6900	ns	0.6278	ns	0.4682
C9ALS vs sALS	*	0.0119	ns	0.9969	ns	0.8065
**Cerebellum**						
CTL vs C9ALS	ns	0.3346	ns	0.0537	ns	0.8275
CTL vs sALS	ns	0.9846	ns	0.0879	ns	0.8328
C9ALS vs sALS	ns	0.4181	ns	0.9614	ns	0.5048
**Motor Cortex**						
CTL vs C9ALS	ns	0.1603	ns	0.9110	ns	0.3868
CTL vs sALS	ns	0.7137	ns	0.9878	ns	0.7339
C9ALS vs sALS	ns	0.4972	ns	0.9627	ns	0.8900
