# Supplementary material for: Dipeptide repeat proteins inhibit homology-directed DNA double strand break repair in C9ORF72 ALS/FTD
Source: Mol Neurodegener. 2020 Feb 24;15:13. doi: 10.1186/s13024-020-00365-9 (PMC7041170; doi:10.1186/s13024-020-00365-9)
Supplement: Supplementary file 8 — Additional file 8. Genome editing eliminates the C9ORF72 hexanucleotide repeat expansion. A) Schematic of editing strategy: adeno-associated viral (AAV) transduction of iPSCs derived from a C9ALS/FTD patient, titration, clonal selection and isolation of genomic DNA for downstream analysis. Location of guide RNAs and PCR primers spanning the HRE. Expected amplicon sizes for the wild type (WT), expanded and edited alleles are indicated. Electrophoresis of PCR amplicons from three iPSC clones C9ALS-1.4 (HRE+/wt-), C9ALS-1.8 (HRE+/wt+), C9ALS-1.11 (HRE−/wt-) using C9 flanking primers (top) or template input control primers (bottom). Sanger sequencing of PCR amplicons from clone C9ALS-1.11 (HRE−/wt-) confirms loss of sequence homology to the reference genome between gRNAs. Schematic of repeat primed PCR amplification of the HRE. Fragment analysis of amplicons for three clones confirms the loss of the HRE in clone C9ALS-1.11. B) Electropherogram output from fragment analysis indicating intensity (Y-axis) and size (X-axis) of amplicons produced by C9ORF72 repeat-primed PCR. Source of template genomic DNA corresponding to each iPSC cell line used in the study is indicated in upper-right corner. Prototypical saw-tooth pattern is evident in three patient derived cell lines with the hexanucleotide expansion (C9ALS-1, C9ALS-4, C9ALS-5) and lack of PCR products in gnomically edited cell lines (iso). analysis of amplicons for all 6 cell lines clones the loss of the HRE in all isogenic clones. C) Representative images of motor neurons from six iPSC lines stained for DAPI (blue) and immunolabeled with antibodies against Tuj1 (green) and PR (red). [file 13024_2020_365_MOESM8_ESM.pdf]

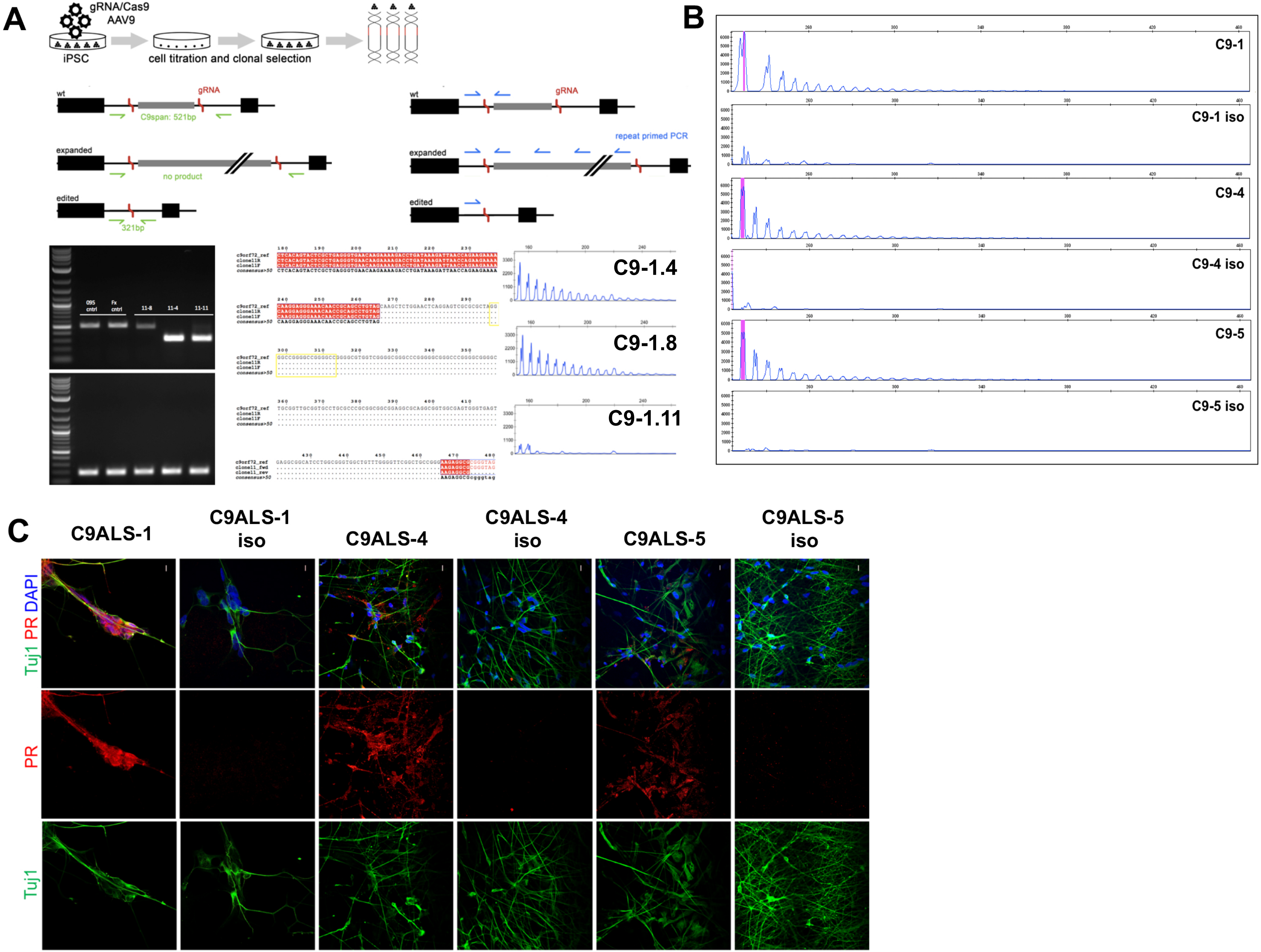
A
gRNA/Cas9
AAV9
iPSC
cell titration and clonal selection
wt
gRNA
C9span: 521bp
expanded
no product
edited
321bp
repeat primed PCR
095 cntrl
Fx cntrl
11-8
11-4
11-11
C9-1.4
C9-1.8
C9-1.11
B
C9-1
C9-1 iso
C9-4
C9-4 iso
C9-5
C9-5 iso
C
C9ALS-1
C9ALS-1 iso
C9ALS-4
C9ALS-4 iso
C9ALS-5
C9ALS-5 iso
Tuj1 PR DAPI
PR
Tuj1
